# Antiplatelet Effects of Flavonoid Aglycones Are Mediated by Activation of Cyclic Nucleotide-Dependent Protein Kinases

**DOI:** 10.3390/ijms25094864

**Published:** 2024-04-29

**Authors:** Anna Balykina, Lidia Naida, Kürsat Kirkgöz, Viacheslav O. Nikolaev, Ekaterina Fock, Michael Belyakov, Anastasiia Whaley, Andrei Whaley, Valentina Shpakova, Natalia Rukoyatkina, Stepan Gambaryan

**Affiliations:** 1 Sechenov Institute of Evolutionary Physiology and Biochemistry of the Russian Academy of Sciences, Saint Petersburg 194223, Russia; balykina.hannah@gmail.com (A.B.); efock@mail.ru (E.F.); anastasiya.ponkratova@yandex.ru (A.W.); natalia.rukoyatkina@gmail.com (N.R.); 2 Faculty of General Medicine, Saint Petersburg State University, Saint Petersburg 199034, Russia; 3 Institute of Biomedical Systems and Biotechnologies, Peter the Great Saint Petersburg Polytechnic University, Saint Petersburg 195251, Russia; nayda.lidiya@mail.ru; 4 Institute of Experimental Cardiovascular Research, University Medical Center Hamburg-Eppendorf, 20251 Hamburg, Germany; kuersat.kirkgoez@stud.uke.uni-hamburg.de (K.K.); v.nikolaev@uke.de (V.O.N.); 5 German Center for Cardiovascular Research (DZHK), Partner Site Hamburg/Kiel/Lübeck, 20246 Hamburg, Germany; 6 Research Institute of Hygiene, Occupational Pathology and Human Ecology, Saint Petersburg 188663, Russia; mihail-belyakov@yandex.ru; 7 Department of Pharmacognosy, Saint Petersburg State Chemical and Pharmaceutical University, Saint Petersburg 197022, Russia; andrey.ueyli@pharminnotech.com; 8 Institute for Cardiovascular and Metabolic Research, School of Biological Sciences, University of Reading, Reading RG6 6AS, UK; spakovavalentina@gmail.com

**Keywords:** antiplatelet therapy, platelets, flavonoids, thromboxane synthase (TxS), cyclic adenosine monophosphate (cAMP), cyclic guanosine monophosphate (cGMP), phosphodiesterase (PDE), PDE inhibitors, protein kinase A (PKA), protein kinase G (PKG)

## Abstract

Flavonoid aglycones are secondary plant metabolites that exhibit a broad spectrum of pharmacological activities, including anti-inflammatory, antioxidant, anticancer, and antiplatelet effects. However, the precise molecular mechanisms underlying their inhibitory effect on platelet activation remain poorly understood. In this study, we applied flow cytometry to analyze the effects of six flavonoid aglycones (luteolin, myricetin, quercetin, eriodictyol, kaempferol, and apigenin) on platelet activation, phosphatidylserine externalization, formation of reactive oxygen species, and intracellular esterase activity. We found that these compounds significantly inhibit thrombin-induced platelet activation and decrease formation of reactive oxygen species in activated platelets. The tested aglycones did not affect platelet viability, apoptosis induction, or procoagulant platelet formation. Notably, luteolin, myricetin, quercetin, and apigenin increased thrombin-induced thromboxane synthase activity, which was analyzed by a spectrofluorimetric method. Our results obtained from Western blot analysis and liquid chromatography–tandem mass spectrometry demonstrated that the antiplatelet properties of the studied phytochemicals are mediated by activation of cyclic nucleotide-dependent signaling pathways. Specifically, we established by using Förster resonance energy transfer that the molecular mechanisms are, at least partly, associated with the inhibition of phosphodiesterases 2 and/or 5. These findings underscore the therapeutic potential of flavonoid aglycones for clinical application as antiplatelet agents.

## 1. Introduction

Platelets are small anucleate cells derived from megakaryocytes, which play a fundamental role in hemostasis [1]. Platelets have a wide array of surface receptors that can be activated by platelet agonists, including thrombin, collagen, ADP, and thromboxane A_2_ (TxA_2_). These stimuli promote platelet activation and aggregation, leading to their adhesion to injured endothelium and the formation of localized thrombi [2].

In the absence of vascular damage, blood vessels release prostacyclin and nitric oxide (NO), which promote a quiescent state of platelets by activating the main mechanisms of platelet inhibition. These signals prevent spontaneous platelet activation via the increase in platelet cyclic adenosine monophosphate (cAMP) and cyclic guanosine monophosphate (cGMP) levels, respectively. Elevated cAMP and cGMP activate corresponding protein kinases, protein kinase A (PKA) and protein kinase G (PKG) [3]. The control of cyclic nucleotide activity is accomplished by phosphodiesterases (PDEs). Platelets contain PDE2A (cGMP-stimulated PDE; hydrolyzes both cGMP and cAMP with similar affinities), PDE3A (cGMP-inhibited PDE; hydrolyzes both, but preferentially cAMP), and PDE5A (specifically degrades cGMP) [3]. Under pathological conditions, such as vascular injury, inflammation, atherosclerosis, and cancer metastasis, the quiescent state of platelets may be disrupted [4,5]. In these scenarios, activated platelets contribute to formation of blood clots and progression of underlying diseases.

Cardiovascular diseases and their prevention in subjects at high risk of cardiovascular events remain the main reasons for the administration of antiplatelet therapy [6]. Dual antiplatelet therapy with aspirin and P_2_Y_12_ receptor antagonists is standard for patients with acute myocardial infarction and ischemic stroke [7,8]. Nevertheless, the following development of drug resistance, hypersensitivity [9,10,11,12], and serious adverse effects, among which are peptic ulcers, gastrointestinal bleeding, and aspirin-induced asthma, are associated with the application of this therapy [13,14,15,16]. Despite these challenges, diverse effects not related to platelet function, and cost-effectiveness, make aspirin a priority medication [17,18]. In this regard, the search for novel compounds with antiplatelet activity and beneficial effects is of particular interest.

Flavonoids are a diverse group of polyphenolic compounds found in plants, responsible for their metabolism, color, and flavor [19,20]. Fruits, vegetables, plant-derived beverages, and honey are the prevalent dietary sources of flavonoids [21,22]. Depending on the presence of sugar residues, flavonoids are classified into aglycones and glycosides. These phytochemicals have become the subject of intense research due to a wide range of beneficial effects on human health, such as antioxidant and anti-inflammatory [23,24,25]. Additionally, flavonoids demonstrate anticancer effects by inhibiting growth and inducing apoptosis in cancer cell lines [26,27,28,29], and have the ability to enhance cell survival in non-tumor cells [30,31,32,33]. Considered safe with a wide therapeutic window [34], flavonoids emerge as promising candidates for medical application, causing particular interest in investigating their biological effects. Recent clinical trials have shown the ability of flavonoids to improve post-COVID-19 olfactory dysfunction, reduce hypercoagulability and symptoms of rheumatoid arthritis [35,36,37].

A growing body of evidence supports the efficacy of flavonoid aglycones in treating thrombosis. Flavonoids isolated from *Leuzea carthamoides*, *Premna foetida*, and *Ginkgo biloba* have demonstrated strong antiplatelet effects [38,39,40]. Previous data indicated inhibitory effects of isolated flavonoid aglycones, including luteolin, myricetin, quercetin, eriodictyol, kaempferol, and apigenin, on platelet activation induced by various agonists [38,41,42,43,44]. Noteworthy, the antiplatelet effect of aglycones was stronger in comparison with the effects exhibited by flavonoid glycosides [35]. In a recent study, myricetin demonstrated an antiplatelet effect over six times more robust than aspirin when assessed under similar conditions [42]. In mouse models, luteolin inhibited mesenteric artery thrombosis and collagen-adrenergic-induced pulmonary thrombosis without affecting coagulation, hemostasis, or platelet production [43]. The antiplatelet effect of aglycones may be mediated by inhibition of immunoreceptor tyrosine-based activation motif (ITAM), protein kinase B (PKB), phospholipase C (PLC), and mitogen-activated protein kinase (MAPK) [43,45].

Limited information exists on the effect of flavonoid aglycones on cyclic nucleotide-related pathways in platelets, although this effect has been observed in diverse models. In a rat model of pentylenetetrazole-induced seizures, luteolin pretreatment suppressed seizure induction and severity of symptoms by activating the PKA pathway [46]. In rat corpus cavernosum smooth muscle cells, the inhibitory effect of flavonoids was mediated by PDE5 inhibition, leading to increased cGMP levels [47]. In addition, flavonoid aglycones may increase the expression and activity of endothelial NO-synthase (eNOS) with a subsequent enhancement of NO generation [48,49,50,51]. On this matter, it remains crucial to clarify whether the antiplatelet effects of flavonoid aglycones are orchestrated by the direct activation of cyclic nucleotide-related inhibitory pathways. As this molecular mechanism emerges as the primary pathway underlying the antiplatelet effect, it may downgrade previously established molecular mechanisms to secondary effects.

In this study, we demonstrated the impact of six flavonoid aglycones (the structures are presented in the Supplementary Materials, Figure S1): luteolin, myricetin, quercetin, eriodictyol, kaempferol, and apigenin, on human platelet activation. These compounds inhibited thrombin-induced platelet activation and the formation of reactive oxygen species (ROS) without affecting platelet viability, apoptosis induction, or the formation of procoagulant platelets. Unexpectedly, luteolin, myricetin, quercetin, and apigenin increased thrombin-induced thromboxane synthase (TxS) activity. We also demonstrated in living cells that flavonoid aglycones suppress platelet activation by amplifying the cyclic nucleotide-related pathways via the inhibition of PDE2 and/or PDE5 activity.

## 2. Results

### 2.1. Flavonoid Aglycones Inhibit Thrombin-Induced Platelet Activation

**Figure 1:**
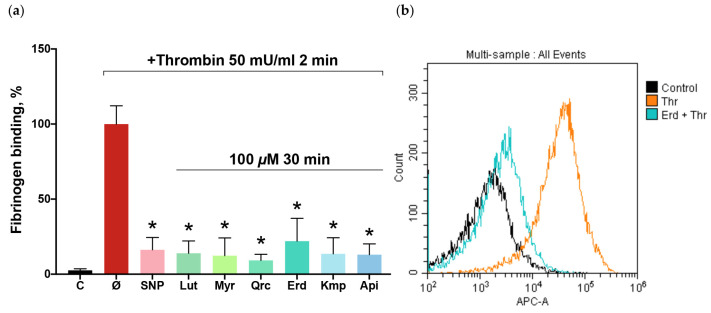
Flavonoid aglycones inhibited thrombin-induced platelet activation. Washed platelets (1 × 10^8^ cells/mL) were incubated with sodium nitroprusside (SNP; 1 μM, 2 min) or the tested flavonoid aglycones (100 μM, 30 min): luteolin (Lut), myricetin (Myr), quercetin (Qrc), eriodictyol (Erd), kaempferol (Kmp), and apigenin (Api). Thrombin (Thr; 50 mU/mL, 2 min) was added to all probes, excluding control (C), and the reaction was stopped by dilution with phosphate buffer (PBS) buffer (1:40). (**a**) Flavonoid aglycones inhibit thrombin-induced platelet *α*IIb*β*3 integrin activation. Data are presented as means ± SD. Thrombin sample was taken as 100%, *n* = 8, non-parametric Mann–Whitney test. *—*p* < 0.05 compared to a thrombin sample. (**b**) The representative histogram (from eight independent experiments) demonstrates the change in thrombin-induced fibrinogen binding to *α*IIb*β*3 integrin when exposed to the effect of eriodictyol.

Flavonoid aglycones are known to inhibit platelet activation induced by different agonists, including thrombin [52]. In experiments with human and rodent platelets, flavonoid aglycones exhibited antiplatelet properties within the concentration range of 2.5–300 μM [53]. Consistent with this, our study showed significant inhibition of sub-threshold thrombin-induced platelet activation by the phytochemicals at a concentration of 100 μM (Figure 1). Among the tested compounds, quercetin demonstrated the highest potency, whereas eriodictyol exhibited the lowest, albeit comparably robust, activity. The observed inhibitory effects of the flavonoid aglycones were comparable to the effect of sodium nitroprusside (SNP), an NO donor, which is known for its strong antiplatelet effect.

### 2.2. Flavonoid Aglycones Reduce ROS Formation in Thrombin-Activated Platelets

**Figure 2:**
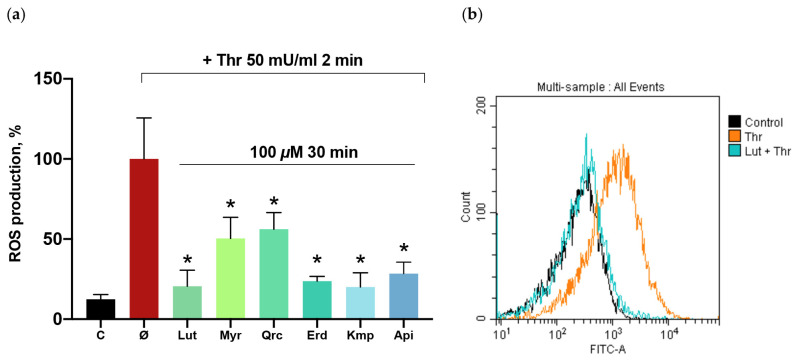
Flavonoid aglycones decreased the level of thrombin-induced ROS formation. Washed platelets (1 × 10^8^ cells/mL) were incubated with DCF-DA (10 μM; 30 min) and the tested flavonoid aglycones (100 μM, 30 min): luteolin (Lut), myricetin (Myr), quercetin (Qrc), eriodictyol (Erd), kaempferol (Kmp), and apigenin (Api). (**a**) Thrombin (Thr; 50 mU/mL, 2 min) was added to all probes, excluding control (C), and the reaction was stopped by dilution with PBS buffer (1:40). Data are presented as means ± SD. The ROS level was designated as 100%, *n* = 6, non-parametric Mann–Whitney test. *—*p* < 0.05 compared to a thrombin sample. (**b**) The representative histogram (from six independent experiments) demonstrates the change in thrombin-induced ROS formation when exposed to the effect of luteolin.

Platelet activation is associated with increased ROS formation [54,55], and flavonoid aglycones demonstrate antioxidant activity in different cell types [56,57]. Nonetheless, it remains unclear whether their effect is related to the ROS formation decrease in platelets. Therefore, we investigated whether the antiplatelet effects of the tested compounds are mediated by the reduction in ROS levels in activated platelets. We employed a cell-permeable DCF-DA dye, which acquires fluorescence upon oxidation by ROS. Our experiments indicated that the aglycones reduce ROS levels in platelets activated by thrombin (Figure 2). Luteolin, eriodictyol, kaempferol, and apigenin reduced ROS levels exceeding 70%, whereas myricetin and quercetin demonstrated a decrease of 50% in ROS formation.

### 2.3. Luteolin, Myricetin, and Quercetin Induce Thromboxane Synthase Activity in Platelets

**Figure 3:**
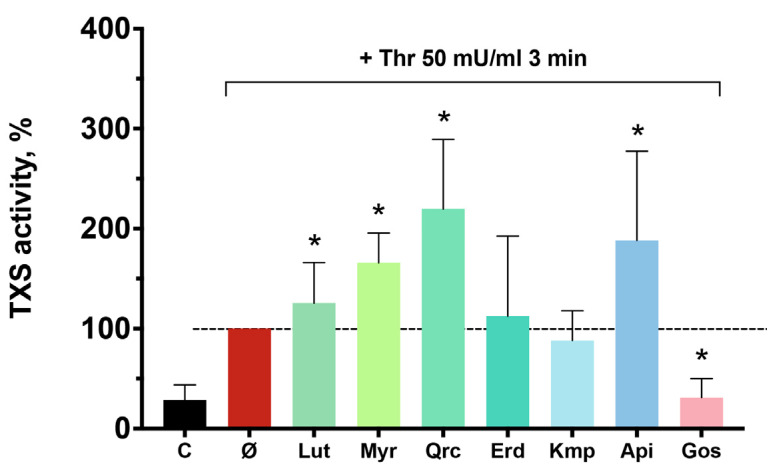
Luteolin, myricetin, quercetin, and apigenin potentiated thrombin-induced thromboxane synthase activity. Washed platelets (5 × 10^8^ cells/mL) were incubated with gossypol (Gos; 40 μM, 10 min) or the indicated compounds (100 μM, 30 min): luteolin (Lut), myricetin (Myr), quercetin (Qrc), eriodictyol (Erd), kaempferol (Kmp), apigenin (Api), in the presence or absence of indomethacin. Thrombin (Thr; 50 mU/mL, 3 min) was added to all probes, excluding control (C), and the reaction was stopped by trichloroacetic acid. The proteins were precipitated, and the level of malondialdehyde (MDA) in the supernatant was estimated from the fluorescence of the product derived from the reaction with thiobarbituric acid. Data are presented as means ± SD. Thrombin sample was taken as 100% and highlighted with a dash line, *n* = 7–9, *t*-test and Wilcoxon signed–ranks test for matched pairs. *—*p* < 0.05 compared to the activator.

Inhibition of thrombin-induced platelet activation may be associated with the reduced release of secondary mediators, such as TxA_2_ [2]. Recent studies have shown that flavonoids can suppress platelet TxA_2_ signaling pathway by binding to TxA_2_ receptors and inhibiting cyclooxygenase-1 (COX-1) [58,59]; however, inhibitory impact of the tested flavonoid aglycones on thrombin-induced TxS activity was not shown before. Due to variable results, we investigated the effect of the phytochemicals on thrombin-induced TxA_2_ synthesis. Unexpectedly, we found that luteolin, myricetin, quercetin, and apigenin at a concentration of 100 μM significantly increase thrombin-induced TxS activity (Figure 3), whereas kaempferol and eriodictyol did not change the activity of TxS in platelets. These results demonstrate that the antiplatelet effects of the tested flavonoids are not connected to the inhibition of TxA_2_ synthesis. On the contrary, luteolin, myricetin, quercetin, and apigenin may potentiate TxS activity through unestablished mechanisms.

### 2.4. Flavonoid Aglycones Do Not Cause Apoptosis or Formation of Procoagulant Phenotype in Platelets

**Figure 4:**
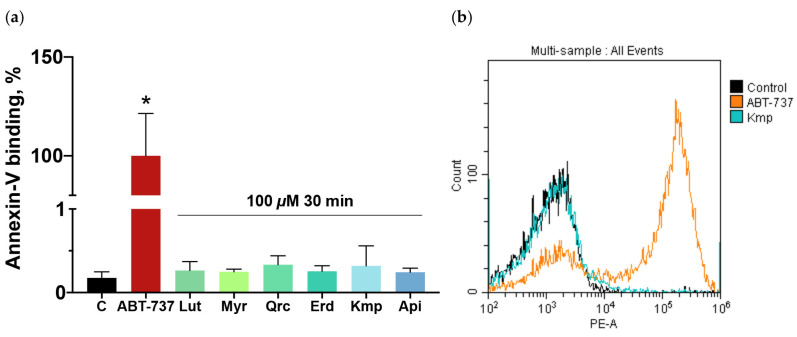
Flavonoid aglycones did not induce phosphatidylserine (PS) exposure in human platelets. Washed platelets (1 × 10^8^ cells/mL) were incubated with the tested flavonoid aglycones (100 μM, 30 min): luteolin (Lut), myricetin (Myr), quercetin (Qrc), eriodictyol (Erd), kaempferol (Kmp), and apigenin (Api). (**a**) Apoptosis inducer ABT-737 (1 μM, 60 min) was used as a positive control. After incubation, Annexin-V-PE (1:10) and Annexin-binding buffer (1:20) were added, then platelets were incubated for 10 min and analyzed by flow cytometry. Data are presented as means ± SD. PS externalization in probes with intact platelets was designated as 100%, *n* = 6, non-parametric Mann–Whitney test. *—*p* < 0.05 compared to an ABT-737 sample. (**b**) The representative histogram (from six independent experiments) demonstrates the change in PS exposure when kaempferol, the flavonoid aglycone with the least anti-apoptotic effect among the tested compounds, was added.

The formation of apoptotic or procoagulant platelets significantly prevents platelet activation and is related to phosphatidylserine (PS) surface exposure [60,61,62]. It was reported that flavonoid aglycones exhibit a proapoptotic effect in cancer cells [63,64] and an anti-apoptotic one in non-cancer cells [65,66]; however, it is not known whether the tested flavonoid aglycones induce the formation of apoptotic or procoagulant platelets. Considering the established link between these platelet phenotypes and inhibition of platelet activation, we evaluated PS exposure on the platelet surface after pre-incubation with the flavonoid aglycones. For positive control, platelets were incubated with an apoptosis inducer, ABT-737 (1 μM, 60 min). The tested flavonoids at a high concentration of 100 μM incubated for 30 min did not induce PS exposure on the outer layer of platelet membrane (Figure 4). Consistent with this, the tested compounds did not cause apoptosis or the formation of a procoagulant phenotype in human platelets.

### 2.5. Flavonoid Aglycones Do Not Affect Platelet Viability

**Figure 5:**
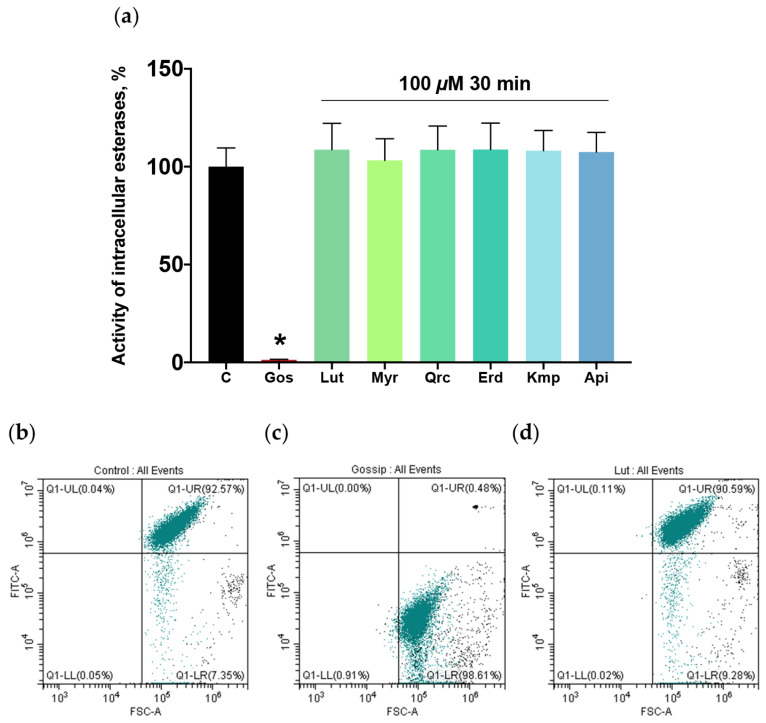
Flavonoid aglycones did not affect platelet viability. Washed platelets (1 × 10^8^ cells/mL) were incubated with the tested flavonoid aglycones (100 μM, 30 min): luteolin (Lut), myricetin (Myr), quercetin (Qrc), eriodictyol (Erd), kaempferol (Kmp), and apigenin (Api). (**a**) Gossypol (Gos; 40 μM, 10 min), which can affect cell viability via inhibition of B-cell lymphoma II (Bcl-2) proteins, was used as a positive control, and the reaction was stopped by dilution with PBS buffer (1:40). Data are presented as means ± SD. Calcein-AM fluorescence in probes with intact platelets was designated as 100%, *n* = 6, non-parametric Mann–Whitney test. *—*p* < 0.05 compared to control. (**b**–**d**) The representative dot plots with platelets pre-selected according to size and granularity marked in green color and background debris in black color (from six independent experiments) demonstrate the activity of intracellular esterases in control (C), gossypol, and luteolin, respectively.

The inhibition of platelet activation can be associated with a reduction in platelet viability. It has been shown that plant extracts rich in flavonoids exhibit significant anti-platelet properties without inducing cytotoxicity [67]. Still, the cytotoxic properties of isolated flavonoid aglycones have not been investigated. Therefore, we evaluated the effect of the flavonoids on platelet viability using a well-established test based on the dye calcein-AM, a fluorogenic substrate of intracellular esterases [68]. For positive control, platelets were incubated with gossypol (40 μM, 10 min), which can affect cell viability via inhibition of B-cell lymphoma II (Bcl-2) proteins. Even at a high concentration of 100 μM, flavonoid aglycones did not affect intracellular esterase activity after 30 min of incubation (Figure 5). These results indicate that the inhibition of platelet activation by the studied phytochemicals is not related to changes in platelet viability.

### 2.6. Flavonoid Aglycones Activate Cyclic Nucleotide-Mediated Signaling Pathways in Platelets

Data concerning the effect of flavonoid aglycones on cyclic nucleotide-related pathways are variable in different cell types [69,70,71,72]. It has not been elucidated before whether the antiplatelet effects of the flavonoid aglycones are mediated by the activation of cyclic nucleotide signaling pathways. Therefore, we investigated the effect of the tested compounds on cAMP/PKA and cGMP/PKG pathways.

**Figure 6:**
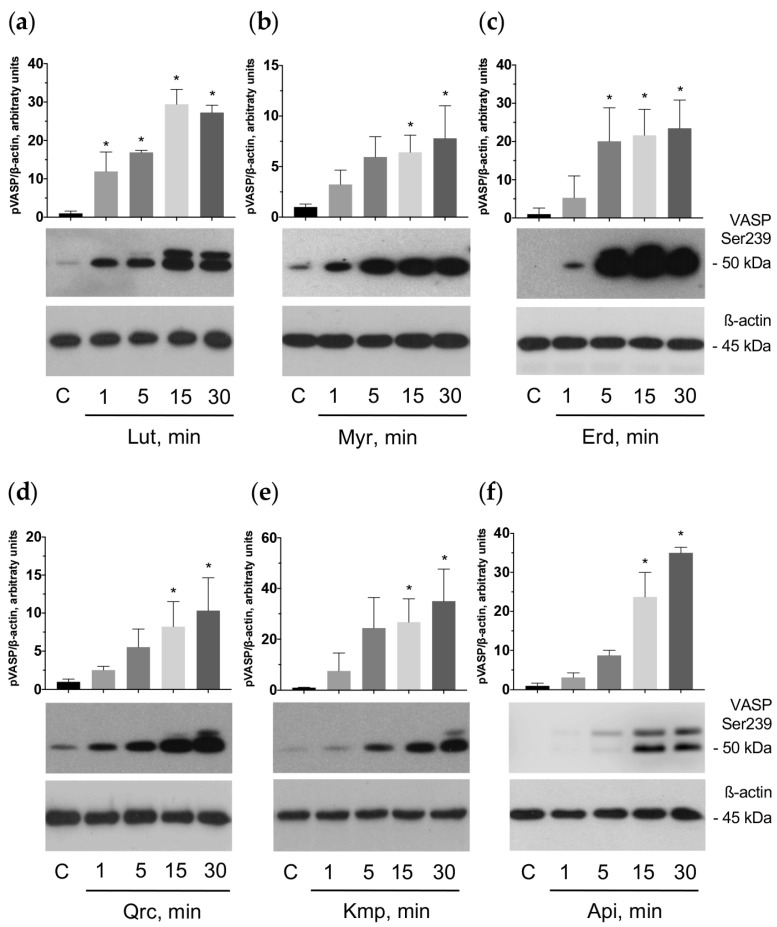
Flavonoid aglycones stimulated VASP phosphorylation time-dependently. Washed human platelets (3 × 10^8^ cells/mL) were incubated with the tested flavonoid aglycones: luteolin (Lut), myricetin (Myr), eriodictyol (Erd), quercetin (Qrc), kaempferol (Kmp), and apigenin (Api) during the indicated time at a concentration of 100 μM (**a**–**f**). Subsequently, probes were lyzed for Western blotting. Actin was used as a loading control. Blots were scanned and quantified by the Image J program. The intensity of the p-VASP signal was normalized to the actin signal. For each sample, this ratio is relatively expressed to the ratio for the control, which is presented as one. Data are presented as means ± SD of three separate experiments from three different donors. One-way ANOVA, Levene’s test *p* > 0.05 followed by Tukey’s HSD test were used for p-VASP. *—*p* < 0.05 compared to control. Representative blots from three independent experiments are shown. Full blots are presented in the Supplementary Materials (Figures S4–S9).

**Figure 7:**
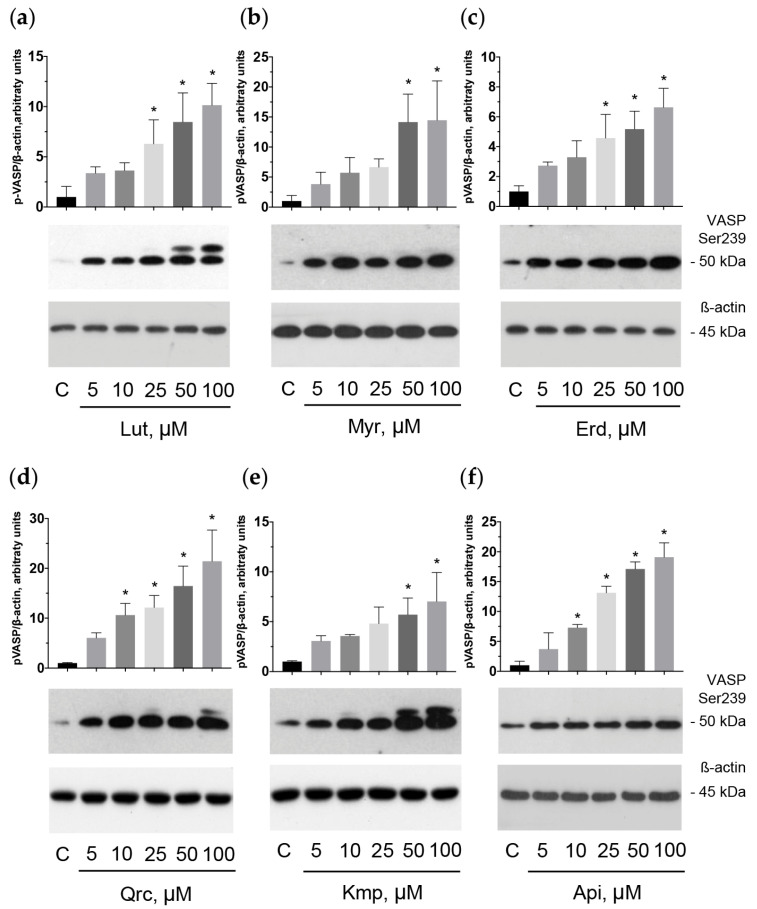
Flavonoid aglycones stimulated VASP phosphorylation dose dependently. Washed human platelets (3 × 10^8^ cells/mL) were incubated with the tested flavonoid aglycones: luteolin (Lut), myricetin (Myr), eriodictyol (Erd), quercetin (Qrc), kaempferol (Kmp), and apigenin (Api) at the indicated concentration for 30 min (**a**–**f**). Subsequently, probes were lyzed for Western blotting. Actin was used as a loading control. Blots were scanned and quantified by the Image J program. The intensity of the p-VASP signal was normalized to the actin signal. For each sample, this ratio is relatively expressed to the ratio for control, which is presented as one. Data are presented as means ± SD of three separate experiments from three different donors. One-way ANOVA, Levene’s test *p* > 0.05 followed by Tukey’s HSD test were used for p-VASP. *—*p* < 0.05 compared to control. Representative blots from three independent experiments are shown. Full blots are presented in the Supplementary Materials (Figures S10–S15).

The vasodilator-stimulated phosphoprotein (VASP) is the major substrate for cyclic nucleotide-related PKA and PKG phosphorylation. PKA preferentially phosphorylates VASP on Ser157, whereas PKG phosphorylates on Ser239; however, strong activation of any kinase leads to the phosphorylation of VASP at both sites [73,74]. Analysis of VASP phosphorylation demonstrated that the antiplatelet effects of the flavonoids are mediated by activation of cyclic nucleotide-related pathways (Figure 6 and Figure 7).

**Figure 8:**
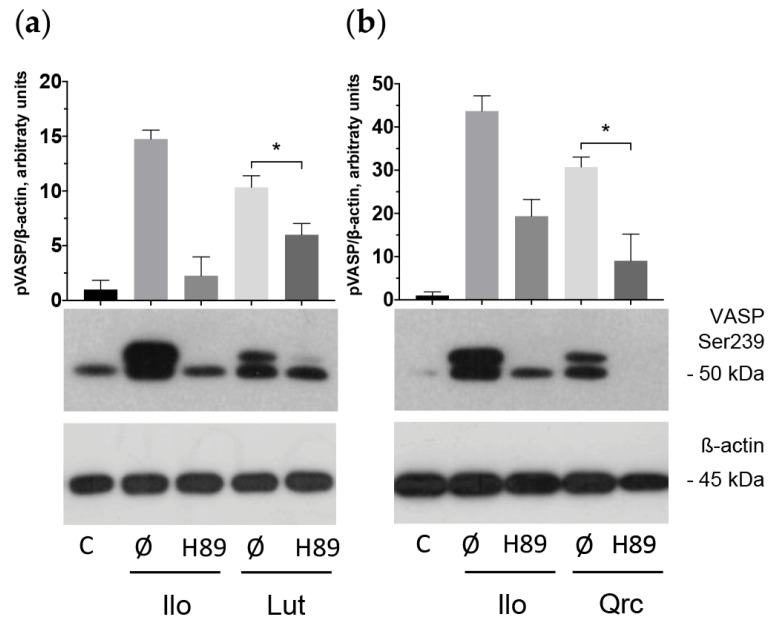
H89 blocked VASP phosphorylation stimulated by the flavonoid aglycones. Washed human platelets (3 × 10^8^ cells/mL) were incubated with H89 (50 μM) for 10 min. Subsequently, some of the tested flavonoid aglycones (100 μM, 30 min): luteolin (Lut) and quercetin (Qrc) were added (**a**,**b**). Iloprost (Ilo; 2 nM, 2 min) was used as a positive control. Probes were lyzed for Western blotting. Actin was used as a loading control. Blots were scanned and quantified by the Image J program. The intensity of the p-VASP signal was normalized to the actin signal. For each sample, this ratio is relatively expressed to the ratio for the control, which is presented as one. Data are presented as means ± SD of three separate experiments from three different donors. One-way ANOVA, Levene’s test *p* > 0.05 followed by Tukey’s HSD test were used for p-VASP. *—*p* < 0.05 compared to a corresponding sample without H89. Representative blots from three independent experiments are shown. Full blots are presented in the Supplementary Materials (Figure S16).

The observed inhibitory effect of these aglycones appeared as time- (1–30 min) and dose-dependent (5–100 μM). In addition, we established that VASP phosphorylation stimulated by the tested aglycones may be significantly suppressed by H89, a specific PKA inhibitor that may also inhibit PKG [75,76] (Figure 8). We concluded that the flavonoid aglycones exhibit antiplatelet effects mediated by the activation of the adenylate cyclase (AC)/cAMP/PKA and/or guanylate cyclase (GC)/cGMP/PKG signaling systems.

### 2.7. Aglycone-Induced Activation of PKA/PKG in Platelets Is Mediated by Inhibition of PDE2 and PDE5

The amplification of cyclic nucleotide-mediated signaling pathways may be due to either activation of AC or GC, or direct activation of PKA or PKG, or inhibition of PDEs. To determine whether the antiplatelet effects of these compounds are related to the activation of AC/GC, we applied inhibitors of AC (SQ22563) and GC (ODQ) prior to incubation with the flavonoid aglycones.

**Figure 9:**
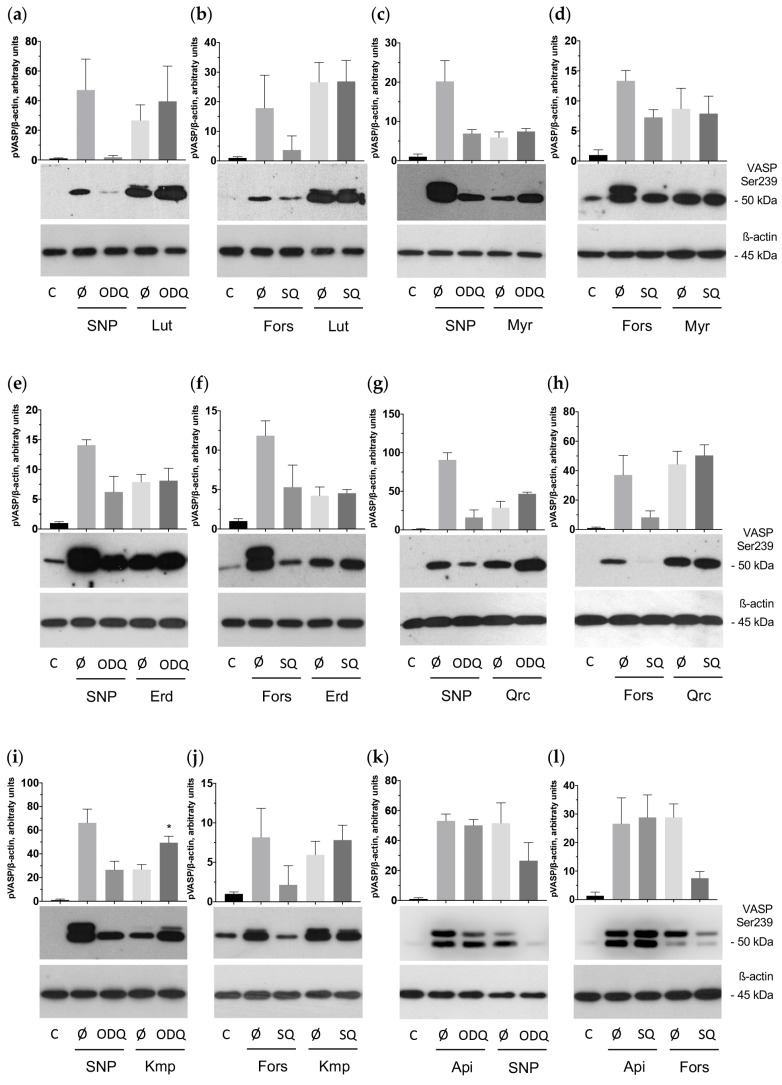
AC and GC inhibitors did not block VASP phosphorylation stimulated by the flavonoid aglycones. Washed human platelets (3 × 10^8^ cells/mL) were incubated for 10 min with ODQ (20 μM) or SQ22563 (100 μM), inhibitors of AC and GC, respectively. Subsequently, the tested flavonoid aglycones (100 μM, 30 min): luteolin (Lut), myricetin (Myr), quercetin (Qrc), eriodictyol (Erd), kaempferol (Kmp), and apigenin (Api) were added (**a**–**l**). Sodium nitroprusside (SNP, 1 μM) and Forskolin (1 μM) were used as positive controls. For Western blotting analysis, probes were lyzed. Actin was used as a loading control. The intensity of the p-VASP signal was normalized to the actin signal. For each sample, this ratio is relatively expressed to the ratio for the control, which is presented as one. Data are presented as means ± SD of three separate experiments from three different donors. One-way ANOVA, Levene’s test *p* > 0.05 followed by Tukey’s HSD test were used for p-VASP. *—*p* < 0.05 compared to a corresponding sample without ODQ or SQ22563. Representative blots from three independent experiments are shown. Full blots are presented in the Supplementary Materials (Figures S16–S22).

**Figure 10:**
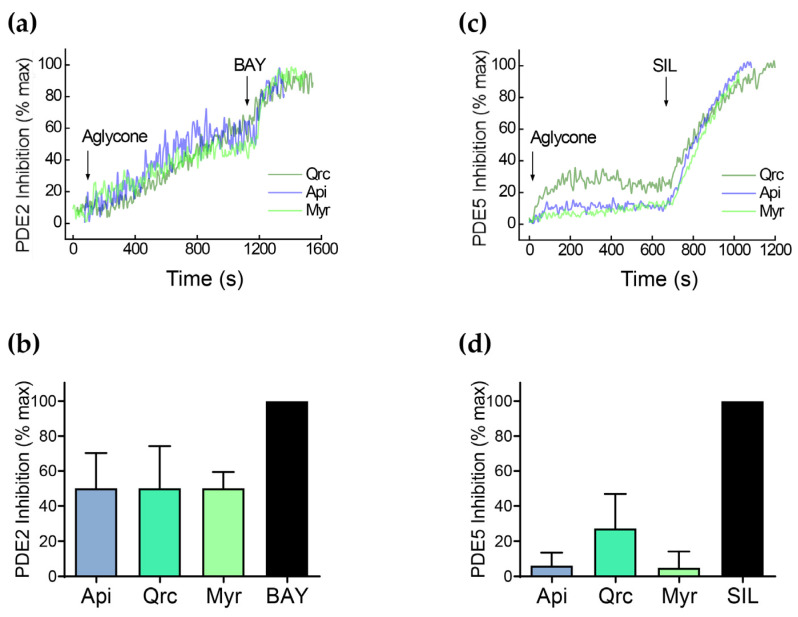
Real-time monitoring of PDE inhibitory activity of flavonoid aglycones in living HEK293 cells expressing Förster resonance energy transfer (FRET)-based biosensors for PDE2 (**a**,**b**) and PDE5 (**c**,**d**) inhibition. Cells pretreated with 50 μM SNP for 10 min were stimulated first with 10 μM of flavonoid aglycones and subsequently with 100 nM BAY 60-7550 or 1 μM sildenafil (SIL) to achieve full inhibition of PDE2 and PDE5, respectively. Representative FRET traces (**a**,**c**) and PDE5 inhibitory response analysis (**b**,**d**) are shown for *n* = 10–20 cells for PDE2 and *n* = 5–10 cells for PDE5.

Our data showed that aglycone-induced VASP phosphorylation is not prevented by inhibition of AC/GC (Figure 9); however, it was slightly and insignificantly decreased by ODQ in the sample containing apigenin. Therefore, we tested whether the activation of PKA/PKG is mediated by the prevention of PDEs activity and the concomitant increase in cAMP/cGMP. The inhibition of different PDEs by several flavonoids used in this study, including some aglycones, was described by in vitro assays [77,78]. To test whether some of the investigated substances (apigenin, quercetin, myricetin) can inhibit PDEs, we used our established live-cell imaging assay for measurement of PDE activity based on Förster resonance energy transfer (FRET) [79] using specific biosensors for PDE2A and PDE5A expressed in HEK293 cells (Figure 10). All three tested aglycones strongly inhibited PDE2 activity, whereas PDE5 was inhibited only moderately. The strongest inhibition of PDE5 was detected by quercetin (Figure 10).

**Figure 11:**
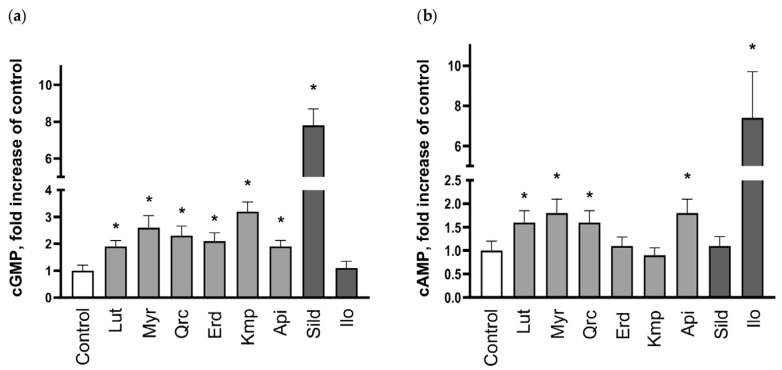
Aglycones increased cyclic nucleotides concentration in platelets. Washed human platelets (3 × 10^8^ cells/mL) were incubated with 100 μM of aglycones for 30 min: luteolin (Lut), myricetin (Myr), quercetin (Qrc), eriodictyol (Erd), kaempferol (Kmp), and apigenin (Api); then, the reaction was stopped by addition of the same volume of 0.2 M HCl. Samples were dried under vacuum, stored at −80 °C for measurement of cAMP/cGMP concentration by LC–MS/MS method. Samples stimulated by sildenafil (Sild; 50 μM, 10 min) and iloprost (Ilo; 2 nM, 2 min) were used as positive controls for cGMP and cAMP, respectively. All tested aglycones significantly increased platelet cGMP concentration (**a**), cAMP was significantly increased only by Lut, Myr, Qrc, and Api (**b**). Data are presented as means ± SD, fold increase compared to control taken as *n* = 5, * significant differences from the control, *p* < 0.05. One-way ANOVA, Levene’s test *p* > 0.05 followed by Tukey’s HSD test were used for p-VASP.

Next, we measured cAMP/cGMP concentrations in platelets incubated with aglycones by the LC–MS/MS method. According to the literature [80,81] and our unpublished data, cAMP and cGMP concentrations measured by ELISA or RIA assays are in the nM range (0.2–1 for cGMP and 5–20 for cAMP in 10^8^ platelets/mL). Our methods demonstrated comparable results with ELISA and RIA methods (0.34 ± 0.15 for cGMP, and 11.6 ± 4.7 for cAMP, means ± SD for 10^8^ platelets/mL). cAMP concentration was increased in the samples incubated with luteolin, myricetin, quercetin, and apigenin; in addition to this, all tested compounds increased cGMP concentration. Serving as positive controls for cAMP and cGMP, respectively, iloprost and sildenafil increased the cyclic nucleotide concentrations by more than 7-fold (Figure 11). The presented data indicate that aglycones induce VASP phosphorylation, which is mediated by the increase in cyclic nucleotide concentrations induced by PDE2 and PDE5 inhibition.

**Figure 12:**
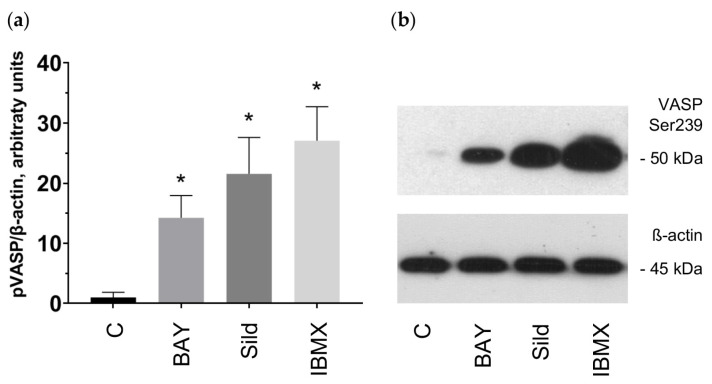
PDE inhibitors stimulated VASP phosphorylation. Washed human platelets (3 × 10^8^ cells/mL) were incubated with PDE inhibitors: BAY 60-7550 (BAY; 5 μM), sildenafil (Sild; 50 μM), IBMX (50 μM) for 10 min (**a**). Subsequently, probes were lyzed for Western blotting. Actin was used as a loading control. Blots were scanned and quantified by the Image J program. The intensity of the p-VASP signal was normalized to the actin signal. For each sample, this ratio is relatively expressed to the ratio for the control, which is presented as one. Data are presented as means ± SD of three separate experiments from three different donors. One-way ANOVA, Levene’s test *p* > 0.05 followed by Tukey’s HSD test were used for p-VASP. *—*p* < 0.05 compared to control. (**b**). A representative blot from three independent experiments is shown. Full blots are presented in the Supplementary Materials (Figure S23).

To confirm our observation about potential VASP stimulation by PDE2/PDE5 inhibitors, we incubated platelets with a PDE2 inhibitor BAY 60-7550, a PDE5 inhibitor sildenafil, and a nonspecific PDE inhibitor IBMX. All three well-known inhibitors of PDEs caused strong VASP phosphorylation (Figure 12) with IBMX showing the most potent effect. These results align with our data concerning the molecular effect of the tested flavonoids.

## 3. Discussion

Flavonoid aglycones exhibit antiplatelet effects, which are partly mediated by the inhibition of platelet activation induced by different agonists [41,78,82]. Consistent with previous studies [42,83], we confirmed that six flavonoid aglycones, including luteolin, myricetin, quercetin, eriodictyol, kaempferol, and apigenin significantly suppress thrombin-induced platelet activation at a concentration of 100 μM. The observed inhibitory effects were comparable to the potent antiplatelet agent SNP, an NO donor, demonstrating the robust inhibitory potential of the tested substances. Prior studies underscored the pivotal role of ROS in platelet αIIbβ3 activation and granule secretion and showed that inhibitors of ROS formation may reduce platelet aggregation [54]. We revealed that the flavonoid aglycones reduce thrombin-induced ROS formation in platelets. This finding aligns with previous studies highlighting the potency of flavonoid aglycones as ROS scavengers [84,85,86].

The TxA_2_ pathway significantly contributes to the amplification of platelet activation. Flavonoids may antagonize TxA_2_ receptors (TP) and inhibit COX-1 activity [53,87,88]; however, the impact of flavonoid aglycones on TxS remains to be addressed. Therefore, we tested whether the antiplatelet effects of the investigated flavonoids are associated with the reduction in thrombin-induced TxS activity in human platelets. Surprisingly, our experiments revealed that eriodictyol and kaempferol do not inhibit thrombin-induced TxS activity, whereas luteolin, myricetin, quercetin, and apigenin contrariwise potentiate TxS activity. This observation underscores the complexity of signaling networks, suggesting the presence of an additional activatory effect of these substances on platelets. However, these results contrast with reports on the antiplatelet effect of onion peel extract and green tea catechins, both containing quercetin and other isolated flavonoid aglycones, showing the inhibitory effect or the absence of an effect on TxS [41,58,59,89]. The discrepancies observed in the results may be attributed to divergent experimental settings and underscore the need for further research.

Several studies have shown that flavonoid aglycones affect platelet activation via inhibition of ITAM, PKB, and PLC activity [43,45], which may be suppressed by activation of cyclic nucleotide-related signaling pathways [90]. However, the link between antiplatelet effects caused by aglycones and activation of these pathways has not been established before. In this study, we examined whether the effects of the flavonoid aglycones are connected to the activation of cyclic nucleotide signaling [91]. We clearly showed that the phytochemicals time- (1–30 min) and dose-dependently (5–100 μM) stimulate VASP phosphorylation, which can be blocked by H89, a specific PKA and, partly, a PKG inhibitor [75]. Hence, the flavonoid aglycones demonstrate antiplatelet effects mediated by activation of cAMP- and/or cGMP-dependent signaling pathways.

The activation of cyclic nucleotide-mediated signaling may stem from various mechanisms, including the stimulation of AC/GC, direct activation of PKA/PKG, or inhibition of PDEs. AC and GC can be activated directly or indirectly via the binding to G-protein-coupled receptors on the platelet surface or the generation of nitric oxide (NO), respectively [3]. In previous studies, we have identified that the antiplatelet effects of such phytochemicals as curcumin and nobiletin are mediated by activation of the adenosine receptor A_2A_ [92,93]. Flavonoid aglycones may also stimulate eNOS [94,95], which underpins NO production; however, there is not sufficient evidence regarding NOS expression in platelets [91]. Therefore, we examined the presence of an activatory effect of the tested compounds on AC/GC. We showed that VASP phosphorylation induced by the flavonoids is not blocked by either the AC inhibitor or the GC inhibitor. Next, we tested whether some of the investigated substances, among which were apigenin, quercetin, and myricetin, inhibit PDEs in platelets. Our presented data indicated that the antiplatelet effects of these aglycones are mediated by robust inhibition of PDE2 and moderate inhibition of PDE5. The subsequent measurement of cAMP/cGMP concentrations in platelets incubated with all tested aglycones provided evidence that the tested compounds elevate cGMP concentration, whereas luteolin, myricetin, quercetin, and apigenin also increase cAMP concentration. Thus, it can be concluded that the tested flavonoids inhibit PDE2 or PDE5, as do apigenin, quercetin, and myricetin, increasing the respective concentrations of cyclic nucleotides. Importantly, a more functional FRET-based assay performed in cells expressing specific PDE2A- and PDE5A-based biosensors confirmed that apigenin, quercetin, and myricetin all strongly inhibit PDE2, and quercetin also strongly inhibits PDE5. In contrast, apigenin and quercetin only slightly inhibited PDE5, at least at 10 μM concentration. The PDE-inhibitory potential of flavonoids was described in a recent review [96], and we originally demonstrated that the antiplatelet effects of flavonoid aglycones are mediated by this mechanism. However, a moderate increase in cyclic nucleotide concentrations did not exclude the direct effect on PKA/PKG and, thus, more research is needed. We also presume that the antioxidant effect of the flavonoids is rather secondary, considering the initially potent activation of PKA/PKG via the inhibition of PDEs [97]. In addition, PDEs are expressed by a variety of cells [97]; therefore, these flavonoid aglycones may have other beneficial effects mediated by PDE inhibition beyond their impact on platelets.

To date, the link between the antiplatelet effects of the isolated flavonoid aglycones and the formation of apoptotic or necrotic platelet phenotypes has not been extensively studied. Addressing concerns related to drug-induced cytotoxicity, we investigated the impact of the tested flavonoid aglycones on platelet viability. In contrast to cancer cells [98,99,100], the phytochemicals, even at a high concentration of 100 μM, did not induce changes in intracellular esterase activity or PS exposure, indicating that their antiplatelet effects are not associated with apoptosis induction, the formation of procoagulant platelets, or cytotoxicity, thus demonstrating a safety profile. The antioxidant and antiplatelet effects found in flavonoid aglycones make them a promising therapeutic option for subjects at risk of thrombosis, especially for elderly individuals [101]. Due to the evidence of antioxidant effects, several flavonoids have also been considered for the treatment of thrombocytopenia [102]. However, here we showed that the tested flavonoid aglycones possess strong antiplatelet effects; thus, elevated risks of bleeding should not be excluded in these patients applying flavonoid aglycones as comedication. Flavonoids also appear as strong anti-apoptotic agents in non-cancer cells [103,104]; consequently, it is essential to investigate the anti-apoptotic effect of the tested compounds in platelets.

**Figure 13:**
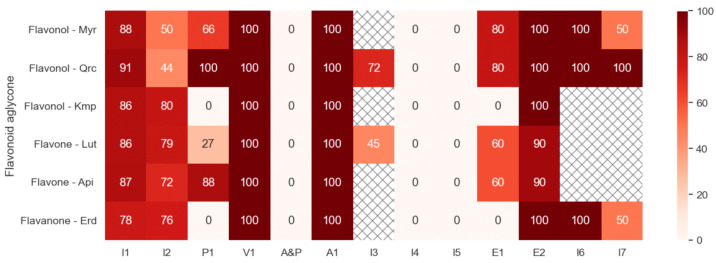
Heatmap of the flavonoid properties identified in this study. Each column represents the tested flavonoid aglycones (100 μM, 30 min): myricetin (Myr), quercetin (Qrc), kaempferol (Kmp), luteolin (Lut), apigenin (Api), and eriodictyol (Erd), which were arranged based on the subclassification depending on their structures (flavonols, flavones, and a flavanone). The rows represent the diverse effects tested within this study: inhibition of thrombin-induced platelet activation (I1), inhibition of thrombin-induced ROS formation (I2), potentiation of thrombin-induced TxS activity (P1), viability of platelets (V1), apoptotic or procoagulant platelet formation (A&P), activation of cyclic nucleotide-related pathways (A1), inhibition of flavonoids’ effect on cyclic nucleotide-related pathways by H89 (I3), inhibition of flavonoids’ effect on AC by SQ22563 (I4), inhibition of flavonoids’ effect on GC by ODQ (I5), elevation of cAMP levels (E1), elevation of cGMP levels (E2), inhibition of PDE2 (I6), inhibition of PDE5 (I7). The intensity of these effects is quantified in relative percentage terms, derived from the corresponding data outlined in the Section 2. The values were quantized to integer numbers and clipped in the range of 0–100 to provide better representation. In case of insignificant changes, the values were replaced with 0 or 100, where relevant. The shaded boxes indicate the absence of experiments. Data are displayed as colors ranging as shown in the key.

To consolidate and visualize the effects of the tested compounds established in the present study, we employed a heatmap analysis (Figure 13). From the heatmap, we surprisingly observed a correlation between the presence of cAMP elevation and the potentiation of thrombin-induced TxS activity by myricetin, quercetin, luteolin, and apigenin. This finding warrants consideration in future studies exploring changes in TxS activity mediated by these flavonoid aglycones. The arrangement of flavonoid aglycones based on their subclassification (flavonols, flavones, and flavanones) did not reveal any structural dependence of effects.

Taken together, our findings demonstrate that some flavonoid aglycones, such as luteolin, myricetin, eriodictyol, quercetin, kaempferol, and apigenin, significantly inhibit platelet activation by different underlying mechanisms. These compounds exhibited inhibitory effects on thrombin-induced platelet activation and ROS formation. We elucidated in living cells that the molecular mechanisms of the antiplatelet effects are mediated by activation of cyclic nucleotide-related pathways caused by inhibition of PDE2 and/or PDE5 activity. Given the safety profile, diverse health benefits, and strong antiplatelet effects, these flavonoid aglycones may be considered an alternative to existing antiplatelet therapies.

## 4. Materials and Methods

### 4.1. Chemicals, Reagents, and Materials

Kaempferol, quercetin, and eriodictyol were isolated from the aerial part of *Impatiens grandulifera* Royle as described before [105]; the purities were 97%, 95%, and 99%, respectively (Figure S2). Luteolin (≥98%), myricetin (≥96.0%), apigenin (≥95.0%), ODQ, SQ22563, gossypol, forskolin, sodium nitroprusside, 3-isobutyl-1-methylxanthine (IBMX), sildenafil, indomethacin, formic acid, H89, iloprost (Sigma-Aldrich, St. Louis, MO, USA); thrombin (Roche, Mannheim, Germany); BAY 60-7550 (Santa Cruz Biotechnology, Heidelberg, Germany); cAMP and cGMP (Merck, Rahway, NJ, USA); acetonitrile (HPLC grade) from ITW Group (Glenview, IL, USA); isopropylic alcohol (Lenreactiv, St. Petersburg, Russia); isotope-labeled cAMP (cAMP-13C5; TRC, North York, ON, Canada), anti-β-actin (# 4970) antibodies (Cell Signaling, Frankfurt, Germany); phospho-VASPS239 (Clone 16c2) (Nano Tools, Teningen, Germany); fibrinogen-Alexa-Fluor 647, calcein-AM (Molecular Probes, Göttingen, Germany); PE-conjugated Annexin-V (BD Bioscience, Heidelberg, Germany); 2′,7′-dichlorodihydrofluorescein diacetate (DCF-DA) (Calbiochem, Schwalbach, Germany), ABT-737 (Selleckchem, Munich, Germany); horseradish peroxidase-conjugated anti-rabbit or anti-mouse IgG (Amersham, Freiburg, Germany), were all utilized in this experiment.

### 4.2. Human Platelet Preparation

The study was conducted in accordance with the Declaration of Helsinki, and all experimental protocols were submitted and approved by the Ethical Committee of Sechenov Institute of Evolutionary Physiology and Biochemistry of the Russian Academy of Sciences (protocol no. 1–04 from 7 April 2022). Signed written consents were obtained prior to venipuncture. Platelets from human eligible, voluntary donors were isolated following established procedures with minor modifications as previously described [106]. Briefly, blood was collected into a citrate Monovette© with Acid Citrate Dextrose (ACD) solution (12 mM citric acid, 15 mM sodium citrate, 25 mM D-glucose) with the addition of EGTA 0.5 M. The whole blood underwent centrifugation at a speed of 1300 RPM for 8 min at RT to obtain platelet-rich plasma (PRP). Subsequently, PRP was centrifuged at 2400 RPM for 4 min, and platelet pellets were washed once with CGS buffer (120 mM sodium chloride, 12.9 mM trisodium citrate, 10 mM D-glucose, pH 6.5), then centrifuged under the same conditions. Finally, washed platelets (WP) were resuspended in HEPES buffer (150 mM sodium chloride, 3 mM potassium chloride, 1 mM magnesium chloride, 5 mM D-glucose, 10 mM HEPES, pH 7.4). Following a 10 min resting period for the platelets at 37 °C, 1 mM CaCl_2_ was added.

### 4.3. Flow Cytometry Analysis

The CytoFLEX flow cytometer (Beckman Coulter, Inc., Brea, CA, USA; instrument at the Center for Collective Use of the Institute of Evolutionary Physiology and Biochemistry of the Russian Academy of Sciences) was used for the experimental analysis. WP concentration of 1 × 10^8^/mL was used, and DMSO was added as a vehicle of the flavonoid aglycones to control samples. A total of 15,000 events were recorded for each sample. Data analysis was performed in CytExpert Acquisition and Analysis Software Version 2.4 (Beckman Coulter, Inc., USA).

#### 4.3.1. Analysis of Platelet αIIbβ3 Integrin Activation

Platelet *α*IIb*β*3 integrin activation was measured by fibrinogen-Alexa-Fluor 647 binding. Fibrinogen (final concentration 15 μg/mL) was added to WP, and platelets were incubated with the flavonoid aglycones (100 μM) at 37 °C for 30 min. After the addition of platelet agonist thrombin (50 mU/mL), the samples were incubated at 37 °C for 2 min. Finally, the reaction was stopped by the addition of PBS (1:40).

#### 4.3.2. Analysis of Phosphatidylserine Exposure

PS exposure was measured by annexin-V-PE binding. Platelets were incubated with flavonoid aglycones (100 μM, 30 min) at 37 °C. Subsequently, annexin-V-PE (1:10) was added to the samples, and the suspension was immediately diluted with Annexin-V binding buffer (140 mM NaCl, 10 mM HEPES, 2.5 mM CaCl_2_). The samples were incubated for 10 min at room temperature (RT) in the dark. For positive control, platelets were incubated with apoptosis inducer ABT-737 (1 μM, 60 min) [60].

#### 4.3.3. Analysis of Platelet Viability

Cell-permeable calcein-AM was used as a marker of platelet viability. Calcein-AM acquires a green fluorescent signal after the acetoxymethyl ester hydrolysis by intracellular esterases [107]. WP were incubated with calcein-AM (0.2 μM) and flavonoid aglycones (100 μM) at 37 °C for 30 min. Then, the reaction was stopped by the addition of PBS (1:40). For positive control, platelets were incubated with gossypol (40 μM, 10 min), which can affect cell viability via inhibition of B-cell lymphoma II (Bcl-2) proteins [108].

#### 4.3.4. Analysis of Reactive Oxygen Species Formation

The analysis of reactive oxygen species (ROS) formation in activated platelets was performed using fluorescent dye DCF-DA. WP were incubated with DCF-DA (10 μM) and flavonoid aglycones (100 μM) at 37 °C for 30 min. Finally, after thrombin (50 mU/mL) incubation at 37 °C for 2 min, the reaction was stopped by PBS (1:40).

### 4.4. Measurement of Thromboxane Synthase Activity

The thromboxane synthase (TxS) activity was evaluated as previously described [109,110]. Briefly, WP (6 × 10^8^ cells/mL) were incubated with gossypol (40 μM; 10 min) or the flavonoid aglycones (100 μM), in the presence or absence of nonselective cyclooxygenase (COX-1 and COX-2) inhibitor indomethacin (10 μM) at 37 °C for 30 min. DMSO was added to control samples as a vehicle of the flavonoid aglycones. Platelets were stimulated with thrombin (50 mU/mL), and the reaction was stopped by trichloroacetic acid (TCA; 20% TCA in 0.6 M HCl). Then, samples were incubated on ice (10 min) and centrifuged (4 °C, 10 min, 4.400 g). The supernatant was mixed with thiobarbituric acid (TBA; 0.53% TBA in 0.01 M KH_2_PO_4_, 0.05 M Na_2_HPO_4_, pH 7.4), heated at 70 °C for 30 min, and cooled at RT. Then, the fluorescence of the reaction product of malondialdehyde (MDA) and TBA was measured (λex = 510 ± 15 nm, λem = 560 ± 20 nm, CLARIOstarPlus reader, BMG Labtech Gmbh, Ortenberg, Germany). To evaluate the TxS activity, the exhibited fluorescence of the samples in the presence of indomethacin was indicated with ethanol (the vehicle for indomethacin).

### 4.5. Western Blot Analysis

WP (3 × 10^8^ cells/mL) were incubated with the flavonoid aglycones at the indicated concentration for the indicated time and lyzed with Laemmli sample buffer. DMSO was added to control samples as a vehicle of flavonoid aglycones. Proteins were separated by SDS polyacrylamide gel (SDS-PAGE) and transferred to nitrocellulose membranes, which were incubated with appropriate primary antibodies overnight at 4 °C. To visualize the signal, either goat anti-rabbit or anti-mouse IgG-conjugated antibodies with horseradish peroxidase were used. ImageJ software version 1.54g (National Institutes of Health, Bethesda, MD, USA, and Laboratory for Optical and Computational Instrumentation, Madison, WI, USA) was used for densitometric analysis.

### 4.6. cAMP and cGMP Measurement

WP (1 × 10^9^ cells/mL) were incubated with the flavonoid aglycones (100 μM) at 37 °C for 30 min. DMSO was added to control samples as a vehicle of flavonoid aglycones. The reaction was stopped by the addition of 0.2 M HCl. Then, the samples were incubated on ice (30 min) and centrifuged (4 °C, 10 min, 20,000× *g*). Samples were dried under vacuum and stored at −80 °C for measurement of cAMP/cGMP concentration by LC–MS/MS method. Samples stimulated by sildenafil (50 μM, 10 min) and iloprost (2 nM, 2 min) were used as positive controls for cGMP and cAMP, respectively.

#### 4.6.1. Liquid Chromatography–Tandem Mass Spectrometry (LC–MS/MS)

Sample extracts were analyzed by HPLC–MS/MS HR system consisting of a Dionex UltiMate 3000 HPLC (Thermo Scientific, Waltham, MA, USA) with Q Exactive detector (Thermo Scientific) with electrospray ionization (ESI). The injection volume of prepared samples and standards is 20 μL. For chromatographic separation, Zorbax SB-C8 150 mm × 4.6 mm × 1.8 μm column was used. The mobile phase was a gradient mixture of two components: solvent A—0.1 M ammonium formiate in water, and solvent B—acetonitrile. The flow rate of the mobile phase was 0.400 mL/min, with the following gradient: 0.0–2.0 min 2% solvent B, then the B content increased to 30% at 8.0 min, and remained so until 9 min, then decreased to 2% at 9.1 min and remained so until the end of the program (11 min). Mass spectrometric detection was performed using negative electrospray ESI (−). The analytes were identified by selecting characteristic target reactions (MRM transitions) and the retention time of the analytes. An example of a chromatogram and mass spectra of the analytes is given in Supplementary Materials (Figure S3). The following MS parameters were kept constant during the analysis: nebulization voltage 4800 V for positive ionization. The temperature of the cone was set at 300 °C, the temperature of the heated probe at 400 °C, the gas flow through the nebulizer at 3 L/min, and the flow rate of the drying gas 10 L/min. Product ions and precursor ions were selected for analyte identification (Table S1).

#### 4.6.2. Preparation of Standard Solutions

To prepare the stock internal standard solution, 10 mg of cAMP-13C5 was accurately weighed (±0.1 mg) using an AUW-220D analytic balance (Shimadzu, Kyoto, Japan), transferred to a 1000 mL volumetric flask and dissolved in 0.1 M HCl in water. Working internal standard solutions (10 ng/mL) were prepared by diluting the stock solutions with 0.1 M HCl in water. Stock solutions were stored at +4 °C for no longer than one week.

To prepare the standard solutions, 10 mg of each substance was accurately weighed (±0.1 mg) using an AUW-220D analytic balance (Shimadzu, Japan), transferred to a 25 mL volumetric flask, and dissolved in the working internal standard solution. Calibration solutions were prepared from the stock solution by dilution with a working internal standard solution. All stock solutions were stored at +4 °C for no longer than one week.

#### 4.6.3. Sample Preparation

A total of 50 μL of the working internal standard solution was added to Eppendorf tubes with samples and thoroughly mixed using a rotary shaker (15 min) and then an ultrasonic unit (15 min). After ultrasonic stirring, the tubes were centrifuged at 14,000 rpm for 5 min. Approximately 40 μL of the supernatant was decanted and transferred into glass vials for HPLC analysis.

### 4.7. Measurements of Phosphodiesterase Inhibition in Living Cells

HEK293 cells were transfected for 24 h using Lipofectamine 2000 to express PDE2A- and PDE5A-cGES-DE5 biosensors designed to measure cGMP hydrolytic activity of these PDEs in intact cells based on FRET [79]. To do so, cells were first prestimulated for 10 min with 50 μM SNP to induce cGMP production followed by 10 μM of flavonoid aglycones (apigenin, myricetin or quercetin) and finally by positive controls—100 nM BAY 60-7550 for PDE2 or 1 μM Sildenafil for PDE5. FRET was monitored using live-cell imaging system built around Leica DMI3000B microscope, DV2 dual-view and optiMOS camera as previously described [111]. Effects of aglycones were calculated as a % of maximal inhibition of the respective PDEs.

### 4.8. Data Analysis

Each dataset represents no less than three different experiments. Data are presented as means ± SD. For data analysis, GraphPad Prism 9 (GraphPad Software, San Diego, CA, USA) was applied. The significance of differences in mean values was determined by the Mann–Whitney U-test for unpaired groups (the data from flow cytometry analysis) and the Wilcoxon signed–ranks test for matched pairs (the data from measurement of thromboxane synthase activity). According to the Shapiro–Wilk test, the data from Western blot analysis and cAMP and cGMP measurement were normally distributed, with Levene’s test *p* > 0.05. Therefore, for group comparisons, one-way ANOVA, and Tukey HSD test was used. Differences between groups were considered statistically significant at *p* < 0.05.

## Data Availability

The data underlying this article will be shared at reasonable request to the corresponding author.

## Supplementary Materials

The supporting information can be downloaded at: https://www.mdpi.com/article/10.3390/ijms25094864/s1.

## References

[1] Jurk K., Kehrel B.E. (2005). Platelets: Physiology and Biochemistry. Semin. Thromb. Hemost..

[2] Bye A.P., Unsworth A.J., Gibbins J.M. (2016). Platelet signaling: A complex interplay between inhibitory and activatory networks. J. Thromb. Haemost..

[3] Smolenski A. (2012). Novel roles of cAMP/cGMP-dependent signaling in platelets. J. Thromb. Haemost..

[4] Mackman N. (2008). Triggers, targets and treatments for thrombosis. Nature.

[5] Franco A.T., Corken A., Ware J. (2015). Platelets at the interface of thrombosis, inflammation, and cancer. Blood.

[6] Iqbal A.M., Lopez R.A., Hai O. (2024). Antiplatelet Medications. StatPearls, StatPearls Publishing Copyright© 2024.

[7] Cosentino N., Campodonico J., Milazzo V., Celentano K., Moltrasio M., Faggiano P., Marenzi G. (2019). Extended dual antiplatelet therapy after acute myocardial infarction. Current evidence and future perspectives. Monaldi Arch. Chest Dis..

[8] Mele F., Gendarini C., Pantoni L. (2023). The use of dual antiplatelet therapy for ischemic cerebrovascular events. Neurol. Sci..

[9] Gasparyan A.Y., Watson T., Lip G.Y. (2008). The role of aspirin in cardiovascular prevention: Implications of aspirin resistance. J. Am. Coll. Cardiol..

[10] Michelson A.D., Frelinger A.L., Furman M.I. (2006). Resistance to antiplatelet drugs. Eur. Heart J. Suppl..

[11] Krasopoulos G., Brister S.J., Beattie W.S., Buchanan M.R. (2008). Aspirin “resistance” and risk of cardiovascular morbidity: Systematic review and meta-analysis. BMJ.

[12] Schwartz K.A. (2011). Aspirin resistance: A clinical review focused on the most common cause, noncompliance. Neurohospitalist.

[13] Bhatt D.L., Cryer B.L., Contant C.F., Cohen M., Lanas A., Schnitzer T.J., Shook T.L., Lapuerta P., Goldsmith M.A., Laine L. (2010). Clopidogrel with or without omeprazole in coronary artery disease. N. Engl. J. Med..

[14] Shiotani A., Sakakibara T., Nomura M., Yamanaka Y., Nishi R., Imamura H., Tarumi K., Kamada T., Hata J., Haruma K. (2010). Aspirin-induced peptic ulcer and genetic polymorphisms. J. Gastroenterol. Hepatol..

[15] Szczeklik A. (2010). Aspirin-induced asthma: A tribute to John Vane as a source of inspiration. Pharmacol. Rep..

[16] Silagy C.A., McNeil J.J., Donnan G.A., Tonkin A.M., Worsam B., Campion K. (1993). Adverse effects of low-dose aspirin in a healthy elderly population. Clin. Pharmacol. Ther..

[17] Aude Y.W., Mehta J.L. (2002). Nonplatelet-mediated effects of aspirin. Drugs Today.

[18] Michelson A.D. (2010). Antiplatelet therapies for the treatment of cardiovascular disease. Nat. Rev. Drug Discov..

[19] Wu J., Lv S., Zhao L., Gao T., Yu C., Hu J., Ma F. (2023). Advances in the study of the function and mechanism of the action of flavonoids in plants under environmental stresses. Planta.

[20] Scarano A., Chieppa M., Santino A. (2018). Looking at Flavonoid Biodiversity in Horticultural Crops: A Colored Mine with Nutritional Benefits. Plants.

[21] Liu J., Wang X., Yong H., Kan J., Jin C. (2018). Recent advances in flavonoid-grafted polysaccharides: Synthesis, structural characterization, bioactivities and potential applications. Int. J. Biol. Macromol..

[22] Petrus K., Schwartz H., Sontag G. (2011). Analysis of flavonoids in honey by HPLC coupled with coulometric electrode array detection and electrospray ionization mass spectrometry. Anal. Bioanal. Chem..

[23] Dias M.C., Pinto D., Silva A.M.S. (2021). Plant Flavonoids: Chemical Characteristics and Biological Activity. Molecules.

[24] Kopustinskiene D.M., Jakstas V., Savickas A., Bernatoniene J. (2020). Flavonoids as Anticancer Agents. Nutrients.

[25] Fraga C.G., Croft K.D., Kennedy D.O., Tomás-Barberán F.A. (2019). The effects of polyphenols and other bioactives on human health. Food Funct..

[26] Imran M., Rauf A., Abu-Izneid T., Nadeem M., Shariati M.A., Khan I.A., Imran A., Orhan I.E., Rizwan M., Atif M. (2019). Luteolin, a flavonoid, as an anticancer agent: A review. Biomed. Pharmacother..

[27] Afroze N., Pramodh S., Hussain A., Waleed M., Vakharia K. (2020). A review on myricetin as a potential therapeutic candidate for cancer prevention. 3 Biotech..

[28] Felice M.R., Maugeri A., De Sarro G., Navarra M., Barreca D. (2022). Molecular Pathways Involved in the Anti-Cancer Activity of Flavonols: A Focus on Myricetin and Kaempferol. Int. J. Mol. Sci..

[29] Li W., Du Q., Li X., Zheng X., Lv F., Xi X., Huang G., Yang J., Liu S. (2020). Eriodictyol Inhibits Proliferation, Metastasis and Induces Apoptosis of Glioma Cells via PI3K/Akt/NF-κB Signaling Pathway. Front. Pharmacol..

[30] Ghorbani A., Rashidi R., Shafiee-Nick R. (2019). Flavonoids for preserving pancreatic beta cell survival and function: A mechanistic review. Biomed. Pharmacother..

[31] Park D.J., Jeon S.J., Kang J.B., Koh P.O. (2020). Quercetin Reduces Ischemic Brain Injury by Preventing Ischemia-induced Decreases in the Neuronal Calcium Sensor Protein Hippocalcin. Neuroscience.

[32] Pei B., Yang M., Qi X., Shen X., Chen X., Zhang F. (2016). Quercetin ameliorates ischemia/reperfusion-induced cognitive deficits by inhibiting ASK1/JNK3/caspase-3 by enhancing the Akt signaling pathway. Biochem. Biophys. Res. Commun..

[33] Boriero D., Carcereri de Prati A., Antonini L., Ragno R., Sohji K., Mariotto S., Butturini E. (2021). The anti-STAT1 polyphenol myricetin inhibits M1 microglia activation and counteracts neuronal death. Febs J..

[34] Markowska A., Antoszczak M., Kacprzak K., Markowska J., Huczyński A. (2023). Role of Fisetin in Selected Malignant Neoplasms in Women. Nutrients.

[35] Zwicker J.I., Schlechter B.L., Stopa J.D., Liebman H.A., Aggarwal A., Puligandla M., Caughey T., Bauer K.A., Kuemmerle N., Wong E. (2019). Targeting protein disulfide isomerase with the flavonoid isoquercetin to improve hypercoagulability in advanced cancer. JCI Insight.

[36] Javadi F., Ahmadzadeh A., Eghtesadi S., Aryaeian N., Zabihiyeganeh M., Rahimi Foroushani A., Jazayeri S. (2017). The Effect of Quercetin on Inflammatory Factors and Clinical Symptoms in Women with Rheumatoid Arthritis: A Double-Blind, Randomized Controlled Trial. J. Am. Coll. Nutr..

[37] Di Stadio A., D’Ascanio L., Vaira L.A., Cantone E., De Luca P., Cingolani C., Motta G., De Riu G., Vitelli F., Spriano G. (2022). Ultramicronized Palmitoylethanolamide and Luteolin Supplement Combined with Olfactory Training to Treat Post-COVID-19 Olfactory Impairment: A Multi-Center Double-Blinded Randomized Placebo-Controlled Clinical Trial. Curr. Neuropharmacol..

[38] Koleckar V., Brojerova E., Rehakova Z., Kubikova K., Cervenka F., Kuca K., Jun D., Hronek M., Opletalova V., Opletal L. (2008). In vitro antiplatelet activity of flavonoids from Leuzea carthamoides. Drug Chem. Toxicol..

[39] Dianita R., Jantan I. (2019). Inhibition of Human Platelet Aggregation and Low-Density Lipoprotein Oxidation by Premna foetida Extract and Its Major Compounds. Molecules.

[40] Chen T.R., Wei L.H., Guan X.Q., Huang C., Liu Z.Y., Wang F.J., Hou J., Jin Q., Liu Y.F., Wen P.H. (2019). Biflavones from Ginkgo biloba as inhibitors of human thrombin. Bioorg Chem..

[41] Tzeng S.H., Ko W.C., Ko F.N., Teng C.M. (1991). Inhibition of platelet aggregation by some flavonoids. Thromb. Res..

[42] Chang Y., Hsia C.W., Huang W.C., Jayakumar T., Hsia C.H., Yen T.L., Sheu J.R., Hou S.M. (2023). Myricetin as a promising inhibitor of platelet fibrinogen receptor in humans. Heliyon.

[43] Ye Y., Yang L., Leng M., Wang Q., Wu J., Wan W., Wang H., Li L., Peng Y., Chai S. (2023). Luteolin inhibits GPVI-mediated platelet activation, oxidative stress, and thrombosis. Front. Pharmacol..

[44] Navarro-Núñez L., Lozano M.L., Palomo M., Martínez C., Vicente V., Castillo J., Benavente-García O., Diaz-Ricart M., Escolar G., Rivera J. (2008). Apigenin inhibits platelet adhesion and thrombus formation and synergizes with aspirin in the suppression of the arachidonic acid pathway. J. Agric. Food Chem..

[45] Gaspar R.S., da Silva S.A., Stapleton J., Fontelles J.L.L., Sousa H.R., Chagas V.T., Alsufyani S., Trostchansky A., Gibbins J.M., Paes A.M.A. (2019). Myricetin, the Main Flavonoid in Syzygium cumini Leaf, Is a Novel Inhibitor of Platelet Thiol Isomerases PDI and ERp5. Front. Pharmacol..

[46] Zhen J.L., Chang Y.N., Qu Z.Z., Fu T., Liu J.Q., Wang W.P. (2016). Luteolin rescues pentylenetetrazole-induced cognitive impairment in epileptic rats by reducing oxidative stress and activating PKA/CREB/BDNF signaling. Epilepsy Behav..

[47] Li J., Wu Y., Yu X., Zheng X., Xian J., Li S., Shi W., Tang Y., Chen Z.S., Liu G. (2022). Isolation, bioassay and 3D-QSAR analysis of 8-isopentenyl flavonoids from Epimedium sagittatum maxim. as PDE5A inhibitors. Chin. Med..

[48] Ferenczyova K., Kalocayova B., Kindernay L., Jelemensky M., Balis P., Berenyiova A., Zemancikova A., Farkasova V., Sykora M., Tothova L. (2020). Quercetin Exerts Age-Dependent Beneficial Effects on Blood Pressure and Vascular Function, But Is Inefficient in Preventing Myocardial Ischemia-Reperfusion Injury in Zucker Diabetic Fatty Rats. Molecules.

[49] Sánchez M., Galisteo M., Vera R., Villar I.C., Zarzuelo A., Tamargo J., Pérez-Vizcaíno F., Duarte J. (2006). Quercetin downregulates NADPH oxidase, increases eNOS activity and prevents endothelial dysfunction in spontaneously hypertensive rats. J. Hypertens..

[50] Taubert D., Berkels R., Klaus W., Roesen R. (2002). Nitric oxide formation and corresponding relaxation of porcine coronary arteries induced by plant phenols: Essential structural features. J. Cardiovasc. Pharmacol..

[51] Zhou Z., Zhou H., Zou X., Wang X., Yan M. (2022). Formononetin regulates endothelial nitric oxide synthase to protect vascular endothelium in deep vein thrombosis rats. Int. J. Immunopathol. Pharmacol..

[52] Beretz A., Cazenave J.P., Anton R. (1982). Inhibition of aggregation and secretion of human platelets by quercetin and other flavonoids: Structure-activity relationships. Agents Actions.

[53] Khan H., Jawad M., Kamal M.A., Baldi A., Xiao J., Nabavi S.M., Daglia M. (2018). Evidence and prospective of plant derived flavonoids as antiplatelet agents: Strong candidates to be drugs of future. Food Chem. Toxicol..

[54] Begonja A.J., Gambaryan S., Geiger J., Aktas B., Pozgajova M., Nieswandt B., Walter U. (2005). Platelet NAD(P)H-oxidase-generated ROS production regulates alphaIIbbeta3-integrin activation independent of the NO/cGMP pathway. Blood.

[55] Salvemini D., Radziszewski W., Mollace V., Moore A., Willoughby D., Vane J. (1991). Diphenylene iodonium, an inhibitor of free radical formation, inhibits platelet aggregation. Eur. J. Pharmacol..

[56] Cruz T.M., Lima A.D.S., Silva A.O., Mohammadi N., Zhang L., Azevedo L., Marques M.B., Granato D. (2023). High-throughput synchronous erythrocyte cellular antioxidant activity and protection screening of phenolic-rich extracts: Protocol validation and applications. Food Chem..

[57] Weng X., Luo X., Dai X., Lv Y., Zhang S., Bai X., Bao X., Wang Y., Zhao C., Zeng M. (2023). Apigenin inhibits macrophage pyroptosis through regulation of oxidative stress and the NF-κB pathway and ameliorates atherosclerosis. Phytother. Res..

[58] Karlíčková J., Říha M., Filipský T., Macáková K., Hrdina R., Mladěnka P. (2016). Antiplatelet Effects of Flavonoids Mediated by Inhibition of Arachidonic Acid Based Pathway. Planta Med..

[59] Son D.J., Cho M.R., Jin Y.R., Kim S.Y., Park Y.H., Lee S.H., Akiba S., Sato T., Yun Y.P. (2004). Antiplatelet effect of green tea catechins: A possible mechanism through arachidonic acid pathway. Prostaglandins Leukot. Essent. Fat. Acids.

[60] Rukoyatkina N., Butt E., Subramanian H., Nikolaev V.O., Mindukshev I., Walter U., Gambaryan S., Benz P.M. (2017). Protein kinase A activation by the anti-cancer drugs ABT-737 and thymoquinone is caspase-3-dependent and correlates with platelet inhibition and apoptosis. Cell Death Dis..

[61] Vogler M., Hamali H.A., Sun X.M., Bampton E.T., Dinsdale D., Snowden R.T., Dyer M.J., Goodall A.H., Cohen G.M. (2011). BCL2/BCL-X(L) inhibition induces apoptosis, disrupts cellular calcium homeostasis, and prevents platelet activation. Blood.

[62] Agbani E.O., Poole A.W. (2017). Procoagulant platelets: Generation, function, and therapeutic targeting in thrombosis. Blood.

[63] Vidya Priyadarsini R., Senthil Murugan R., Maitreyi S., Ramalingam K., Karunagaran D., Nagini S. (2010). The flavonoid quercetin induces cell cycle arrest and mitochondria-mediated apoptosis in human cervical cancer (HeLa) cells through p53 induction and NF-κB inhibition. Eur. J. Pharmacol..

[64] Yuan C., Chen G., Jing C., Liu M., Liang B., Gong G., Yu M. (2022). Eriocitrin, a dietary flavonoid suppressed cell proliferation, induced apoptosis through modulation of JAK2/STAT3 and JNK/p38 MAPKs signaling pathway in MCF-7 cells. J. Biochem. Mol. Toxicol..

[65] Zhang L., Zhou T., Ji Q., He L., Lan Y., Ding L., Li L., Wang Z. (2023). Myricetin improves apoptosis after ischemic stroke via inhibiting MAPK-ERK pathway. Mol. Biol. Rep..

[66] Jiang H., Lin C., Cai T., Jiang L., Lou C., Lin S., Wang W., Yan Z., Pan X., Xue X. (2024). Taxifolin-mediated Nrf2 activation ameliorates oxidative stress and apoptosis for the treatment of glucocorticoid-induced osteonecrosis of the femoral head. Phytother. Res..

[67] Rywaniak J., Luzak B., Podsedek A., Dudzinska D., Rozalski M., Watala C. (2015). Comparison of cytotoxic and anti-platelet activities of polyphenolic extracts from Arnica montana flowers and Juglans regia husks. Platelets.

[68] Hartley P.S., Savill J., Brown S.B. (2006). The death of human platelets during incubation in citrated plasma involves shedding of CD42b and aggregation of dead platelets. Thromb. Haemost..

[69] Pavan B., Capuzzo A., Forlani G. (2015). Quercetin and quercetin-3-O-glucoside interact with different components of the cAMP signaling cascade in human retinal pigment epithelial cells. Life Sci..

[70] Uto T., Ohta T., Yamashita A., Fujii S., Shoyama Y. (2019). Liquiritin and Liquiritigenin Induce Melanogenesis via Enhancement of p38 and PKA Signaling Pathways. Medicines.

[71] Akintunde J.K., Akintola T.E., Aliu F.H., Fajoye M.O., Adimchi S.O. (2020). Naringin regulates erectile dysfunction by abolition of apoptosis and inflammation through NOS/cGMP/PKG signalling pathway on exposure to Bisphenol-A in hypertensive rat model. Reprod. Toxicol..

[72] Cao J., Qiu X., Gao Y., Cai L. (2021). Puerarin promotes the osteogenic differentiation of rat dental follicle cells by promoting the activation of the nitric oxide pathway. Tissue Cell.

[73] Butt E., Abel K., Krieger M., Palm D., Hoppe V., Hoppe J., Walter U. (1994). cAMP- and cGMP-dependent protein kinase phosphorylation sites of the focal adhesion vasodilator-stimulated phosphoprotein (VASP) in vitro and in intact human platelets. J. Biol. Chem..

[74] Smolenski A., Bachmann C., Reinhard K., Hönig-Liedl P., Jarchau T., Hoschuetzky H., Walter U. (1998). Analysis and regulation of vasodilator-stimulated phosphoprotein serine 239 phosphorylation in vitro and in intact cells using a phosphospecific monoclonal antibody. J. Biol. Chem..

[75] Burkhardt M., Glazova M., Gambaryan S., Vollkommer T., Butt E., Bader B., Heermeier K., Lincoln T.M., Walter U., Palmetshofer A. (2000). KT5823 inhibits cGMP-dependent protein kinase activity in vitro but not in intact human platelets and rat mesangial cells. J. Biol. Chem..

[76] Shpakova V., Rukoyatkina N., Walter U., Gambaryan S. (2022). Potential and limitations of PKA/PKG inhibitors for platelet studies. Platelets.

[77] Ko W.C., Shih C.M., Lai Y.H., Chen J.H., Huang H.L. (2004). Inhibitory effects of flavonoids on phosphodiesterase isozymes from guinea pig and their structure-activity relationships. Biochem. Pharmacol..

[78] Zhou Y., Zhang D., Tan P., Xian B., Jiang H., Wu Q., Huang X., Zhang P., Xiao X., Pei J. (2023). Mechanism of platelet activation and potential therapeutic effects of natural drugs. Phytomedicine.

[79] Herget S., Lohse M.J., Nikolaev V.O. (2008). Real-time monitoring of phosphodiesterase inhibition in intact cells. Cell Signal.

[80] Signorello M.G., Leoncini G. (2016). Regulation of cAMP Intracellular Levels in Human Platelets Stimulated by 2-Arachidonoylglycerol. J. Cell Biochem..

[81] Kobsar A., Koessler J., Kehrer L., Gambaryan S., Walter U. (2012). The thrombin inhibitors hirudin and Refludan(^®^) activate the soluble guanylyl cyclase and the cGMP pathway in washed human platelets. Thromb. Haemost..

[82] Guerrero J.A., Lozano M.L., Castillo J., Benavente-García O., Vicente V., Rivera J. (2005). Flavonoids inhibit platelet function through binding to the thromboxane A2 receptor. J. Thromb. Haemost..

[83] Kaneider N.C., Mosheimer B., Reinisch N., Patsch J.R., Wiedermann C.J. (2004). Inhibition of thrombin-induced signaling by resveratrol and quercetin: Effects on adenosine nucleotide metabolism in endothelial cells and platelet-neutrophil interactions. Thromb. Res..

[84] Di Meo F., Lemaur V., Cornil J., Lazzaroni R., Duroux J.L., Olivier Y., Trouillas P. (2013). Free radical scavenging by natural polyphenols: Atom versus electron transfer. J. Phys. Chem. A.

[85] Hassanpour S.H., Doroudi A. (2023). Review of the antioxidant potential of flavonoids as a subgroup of polyphenols and partial substitute for synthetic antioxidants. Avicenna J. Phytomed.

[86] Slika H., Mansour H., Wehbe N., Nasser S.A., Iratni R., Nasrallah G., Shaito A., Ghaddar T., Kobeissy F., Eid A.H. (2022). Therapeutic potential of flavonoids in cancer: ROS-mediated mechanisms. Biomed. Pharmacother..

[87] Guerrero J.A., Navarro-Nuñez L., Lozano M.L., Martínez C., Vicente V., Gibbins J.M., Rivera J. (2007). Flavonoids inhibit the platelet TxA(2) signalling pathway and antagonize TxA(2) receptors (TP) in platelets and smooth muscle cells. Br. J. Clin. Pharmacol..

[88] Zaragozá C., Álvarez-Mon M., Zaragozá F., Villaescusa L. (2022). Flavonoids: Antiplatelet Effect as Inhibitors of COX-1. Molecules.

[89] Ro J.Y., Ryu J.H., Park H.J., Cho H.J. (2015). Onion (*Allium cepa* L.) peel extract has anti-platelet effects in rat platelets. Springerplus.

[90] Huang J., Zhou H., Mahavadi S., Sriwai W., Murthy K.S. (2007). Inhibition of Galphaq-dependent PLC-beta1 activity by PKG and PKA is mediated by phosphorylation of RGS4 and GRK2. Am. J. Physiol. Cell Physiol..

[91] Gambaryan S. (2022). The Role of NO/sGC/cGMP/PKG Signaling Pathway in Regulation of Platelet Function. Cells.

[92] Shpakova V.S., Avdeeva A.V., Al. Arawe N., Prilepskaya A.M., Gambaryan S.P., Alekseeva E.S., Rukoyatkina N.I. (2021). Antiplatelet Effect of Nobiletin is Mediated by Activation of A2A Adenosine Receptor. Biochem. Suppl. Ser. A Membr. Cell Biol..

[93] Rukoyatkina N., Shpakova V., Bogoutdinova A., Kharazova A., Mindukshev I., Gambaryan S. (2022). Curcumin by activation of adenosine A2A receptor stimulates protein kinase a and potentiates inhibitory effect of cangrelor on platelets. Biochem. Biophys. Res. Commun..

[94] Si H., Wyeth R.P., Liu D. (2014). The flavonoid luteolin induces nitric oxide production and arterial relaxation. Eur. J. Nutr..

[95] Cho J.M., Chang S.Y., Kim D.B., Needs P.W., Jo Y.H., Kim M.J. (2012). Effects of physiological quercetin metabolites on interleukin-1β-induced inducible NOS expression. J. Nutr. Biochem..

[96] Orhan I.E., Rauf A., Saleem M., Khalil A.A. (2022). Natural Molecules as Talented Inhibitors of Nucleotide Pyrophosphatases/Phosphodiesterases (PDEs). Curr. Top. Med. Chem..

[97] Gegenbauer K., Elia G., Blanco-Fernandez A., Smolenski A. (2012). Regulator of G-protein signaling 18 integrates activating and inhibitory signaling in platelets. Blood.

[98] Yang C., Song J., Hwang S., Choi J., Song G., Lim W. (2021). Apigenin enhances apoptosis induction by 5-fluorouracil through regulation of thymidylate synthase in colorectal cancer cells. Redox Biol..

[99] Debnath S., Sarkar A., Mukherjee D.D., Ray S., Mahata B., Mahata T., Parida P.K., Das T., Mukhopadhyay R., Ghosh Z. (2022). Eriodictyol mediated selective targeting of the TNFR1/FADD/TRADD axis in cancer cells induce apoptosis and inhibit tumor progression and metastasis. Transl. Oncol..

[100] Tavsan Z., Kayali H.A. (2019). Flavonoids showed anticancer effects on the ovarian cancer cells: Involvement of reactive oxygen species, apoptosis, cell cycle and invasion. Biomed. Pharmacother..

[101] Fuentes E., Palomo I. (2016). Role of oxidative stress on platelet hyperreactivity during aging. Life Sci..

[102] Munir S., Liu Z.W., Tariq T., Rabail R., Kowalczewski P., Lewandowicz J., Blecharczyk A., Abid M., Inam-Ur-Raheem M., Aadil R.M. (2022). Delving into the Therapeutic Potential of Carica papaya Leaf against Thrombocytopenia. Molecules.

[103] Muhammed T.M., Jalil A.T., Taher W.M., Aminov Z., Alsaikhan F., Ramírez-Coronel A.A., Ramaiah P., Farhood B. (2024). The Effects of Apigenin in the Treatment of Diabetic Nephropathy: A Systematic Review of Non-clinical Studies. Mini Rev. Med. Chem..

[104] Ijaz M.U., Nadeem N., Hamza A., Almutairi M.H., Atique U. (2024). Didymin protects against polystyrene nanoplastic-induced hepatic damage in male albino rats by modulation of Nrf-2/Keap-1 pathway. Braz. J. Med. Biol. Res..

[105] Whaley A.K., Lukashov R.I., Whaley A.O., Zhokhova E.V., Gurina N.S., Goncharov M.U., Yakovlev G.P., Tsiarletskaya V.A. (2023). Flavonoids from Impatiens grandulifera and their antioxidant activity. Drug Dev. Regist..

[106] Gambaryan S., Kobsar A., Rukoyatkina N., Herterich S., Geiger J., Smolenski A., Lohmann S.M., Walter U. (2010). Thrombin and collagen induce a feedback inhibitory signaling pathway in platelets involving dissociation of the catalytic subunit of protein kinase A from an NFkappaB-IkappaB complex. J. Biol. Chem..

[107] Wisgrill L., Lamm C., Hartmann J., Preißing F., Dragosits K., Bee A., Hell L., Thaler J., Ay C., Pabinger I. (2016). Peripheral blood microvesicles secretion is influenced by storage time, temperature, and anticoagulants. Cytom. A.

[108] Shpakova V., Rukoyatkina N., Al Arawe N., Prilepskaya A., Kharazova A., Sharina I., Gambaryan S., Martin E. (2022). ML355 Modulates Platelet Activation and Prevents ABT-737 Induced Apoptosis in Platelets. J. Pharmacol. Exp. Ther..

[109] Ledergerber D., Hartmann R.W. (1995). Development of a screening assay for the in vitro evaluation of thromboxane A2 synthase inhibitors. J. Enzym. Inhib..

[110] Aktas B., Utz A., Hoenig-Liedl P., Walter U., Geiger J. (2003). Dipyridamole enhances NO/cGMP-mediated vasodilator-stimulated phosphoprotein phosphorylation and signaling in human platelets: In vitro and in vivo/ex vivo studies. Stroke.

[111] Skryabin E.B., De Jong K.A., Subramanian H., Bork N.I., Froese A., Skryabin B.V., Nikolaev V.O. (2023). CRISPR/Cas9 Knock-Out in Primary Neonatal and Adult Cardiomyocytes Reveals Distinct cAMP Dynamics Regulation by Various PDE2A and PDE3A Isoforms. Cells.

