# Supplementary material for: Antiplatelet Effects of Flavonoid Aglycones Are Mediated by Activation of Cyclic Nucleotide-Dependent Protein Kinases
Source: Int J Mol Sci. 2024 Apr 29;25(9):4864. doi: 10.3390/ijms25094864 (PMC11084604; doi:10.3390/ijms25094864)

Figure S1
Structure of the tested compounds

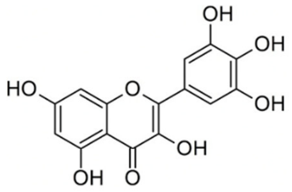

Myricetin

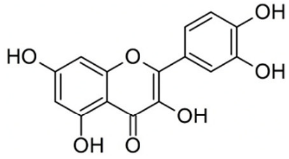

Quercetin

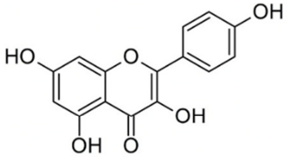

Kaempferol

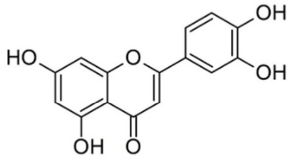

Luteolin

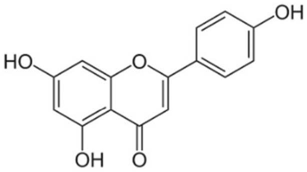

Apigenin

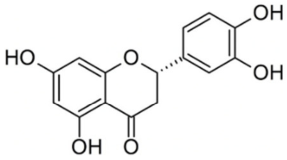

Eriodictyol

Figure S2
High-performance liquid chromatography (HPLC) chromatograms of the isolated compounds: 1 – kaempferol; 2 – quercetin; 3 – eriodictyol

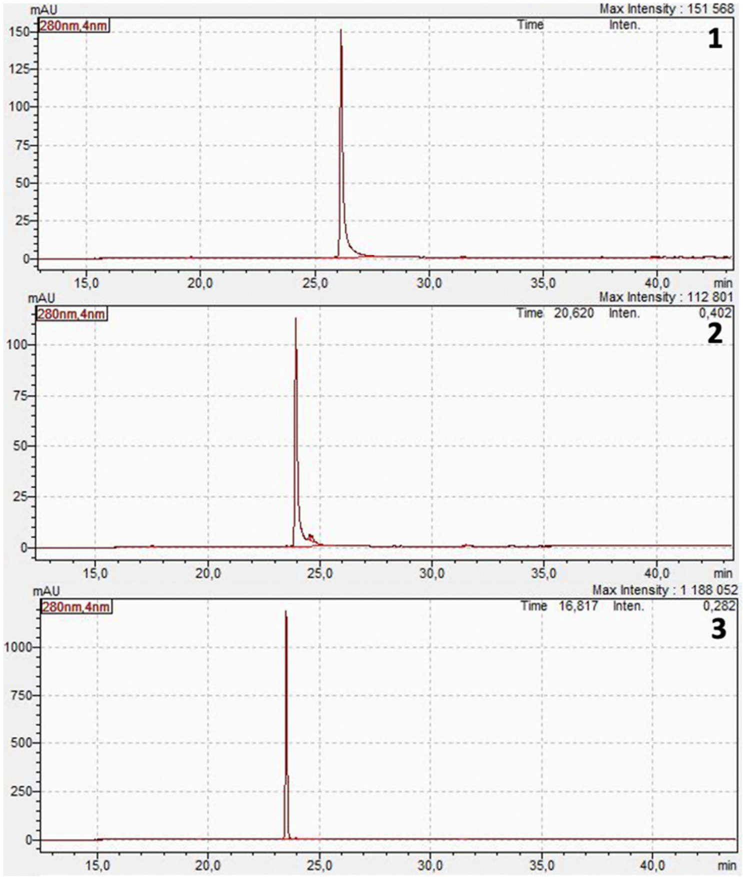

Figure S2. The purity of isolated compounds was 97%, 95% and 99% for kaempferol, quercetin and eriodictyol respectively. The structure of the isolated compounds was elucidated by NMR-spectroscopy. The purity of the compounds was estimated by HPLC using a Prominence LC-20 with a SPD-M20A diode-array detector (Shimadzu corp., Japan) with a Supelcosil LC18 column (250 4.6, 5 lm). The flow rate was 1 mL/min. Analysis temperature - 40 °C. Eluent: water (component A), acetonitrile (component B) with a TFA content of 0.1% (from H_2O : CH_3CN 5 : 95 to H_2O: CH_3CN 0 : 100, by volume). HPLC grade solvents used for HPLC analysis was J.T. Baker HPLC gradient grade.

Figure S3
Mass-chromatogram of sample 3
1 – cGMP, 2 – internal standard, 3 – cAMP.

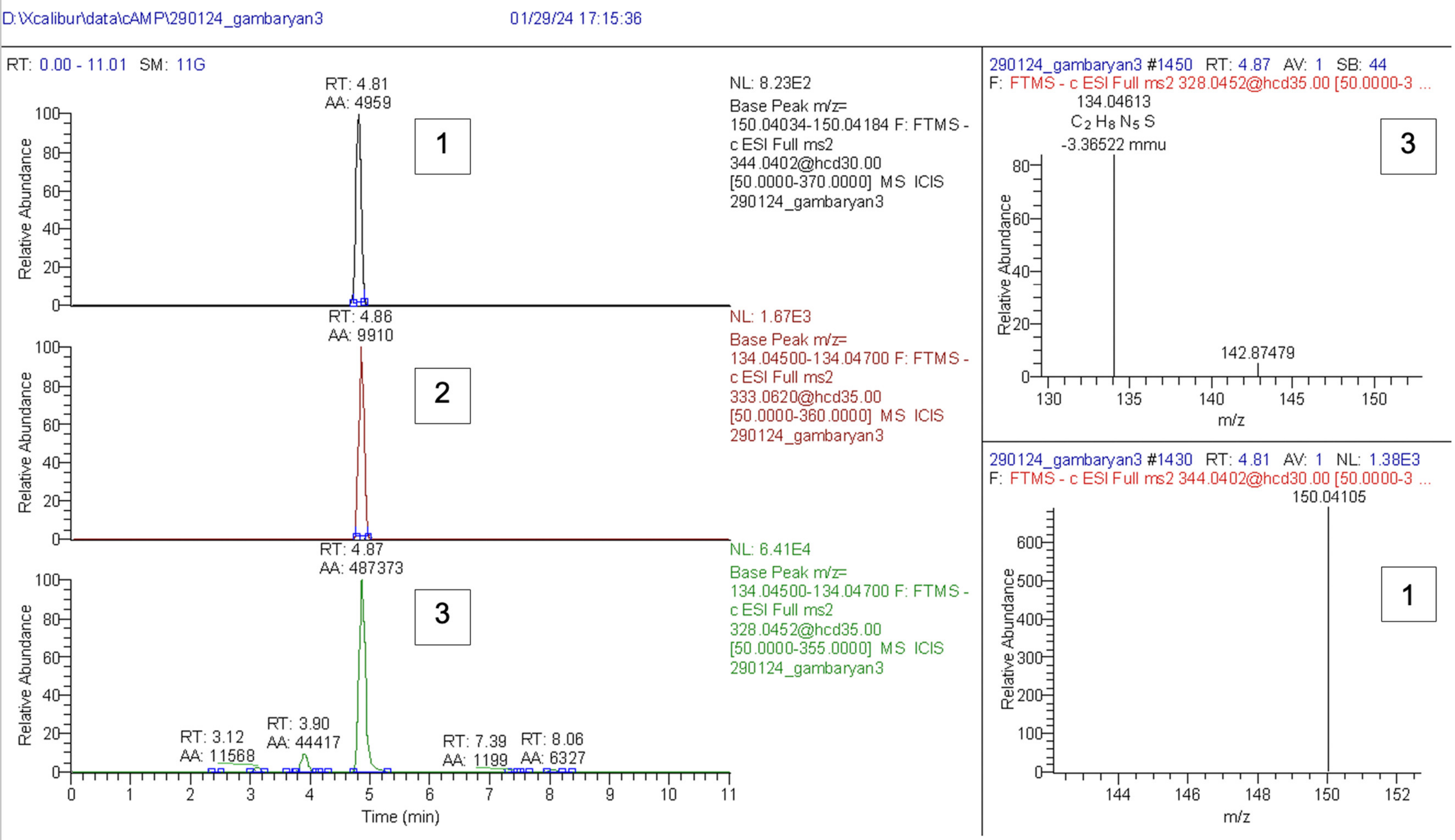

Table S1
Quantification of the cyclic nucleotides
Retention time (tR), multiple reaction monitoring (MRM) employed for identification and quantification of the compounds and internal standard (IS).

Compound	t_R (min)	MRM1
cAMP	4.87	328.0452 => 134.0457
cGMP	4.81	344.0402 => 150.04109
cAMP-13C5 (IS)	4.87	333.0620 => 134.0457

Figure S4
Full blots of figure 6a

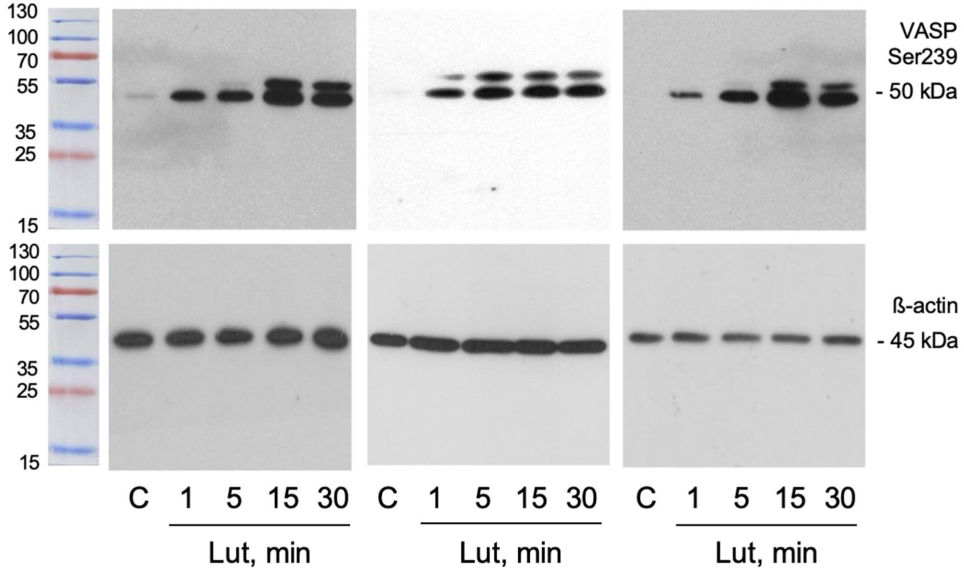

Figure S5
Full blots of figure 6b

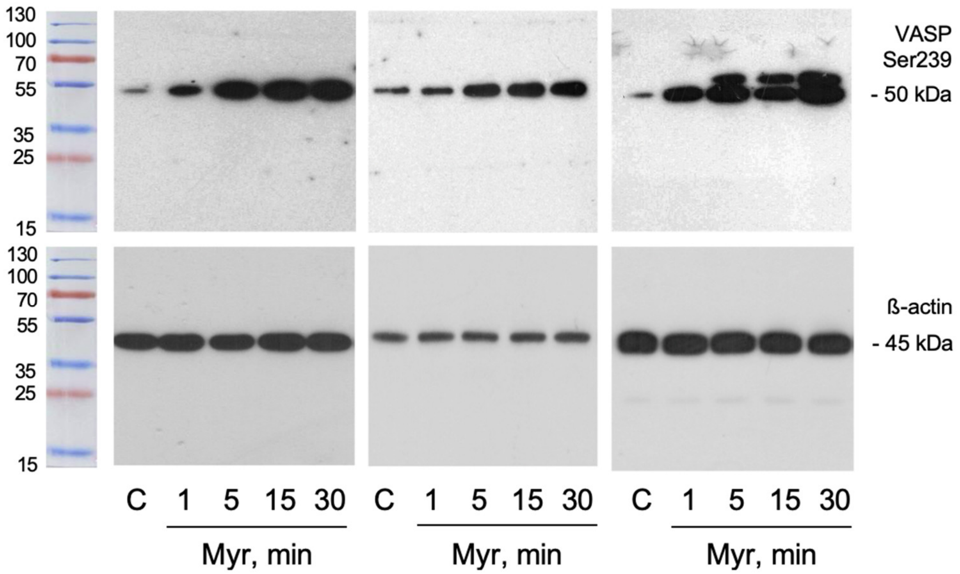

Figure S6
Full blots of figure 6c

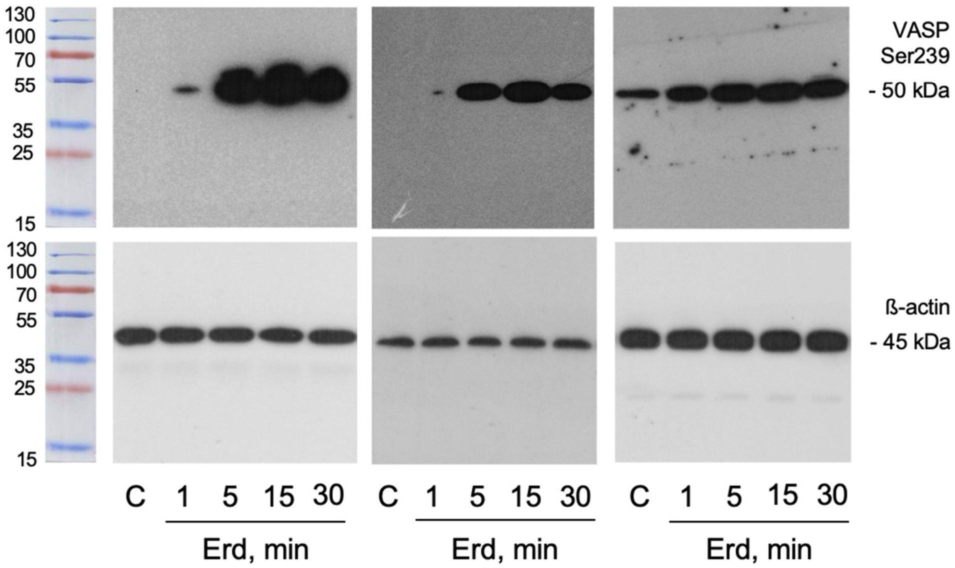

Figure S7
Full blots of figure 6d

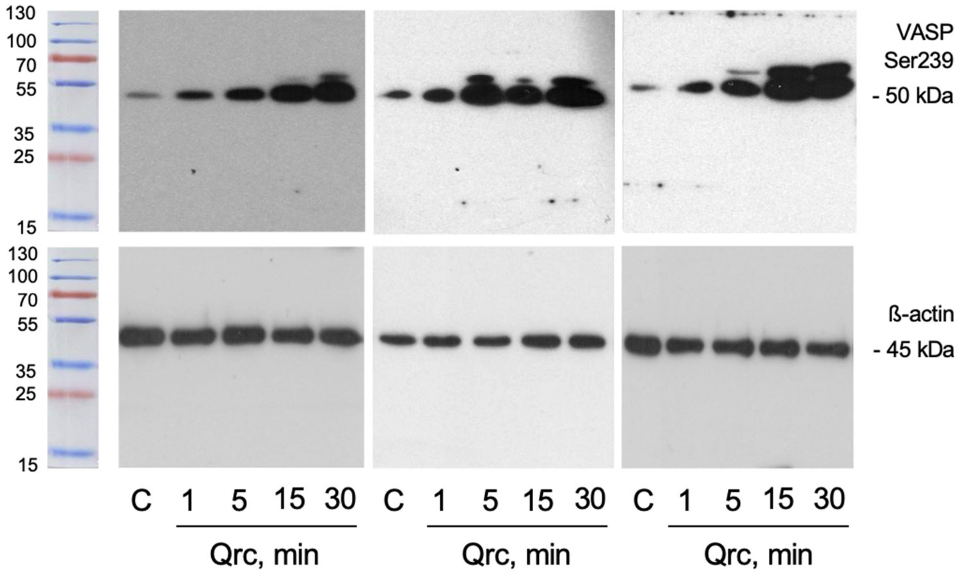

Figure S8
Full blots of figure 6e

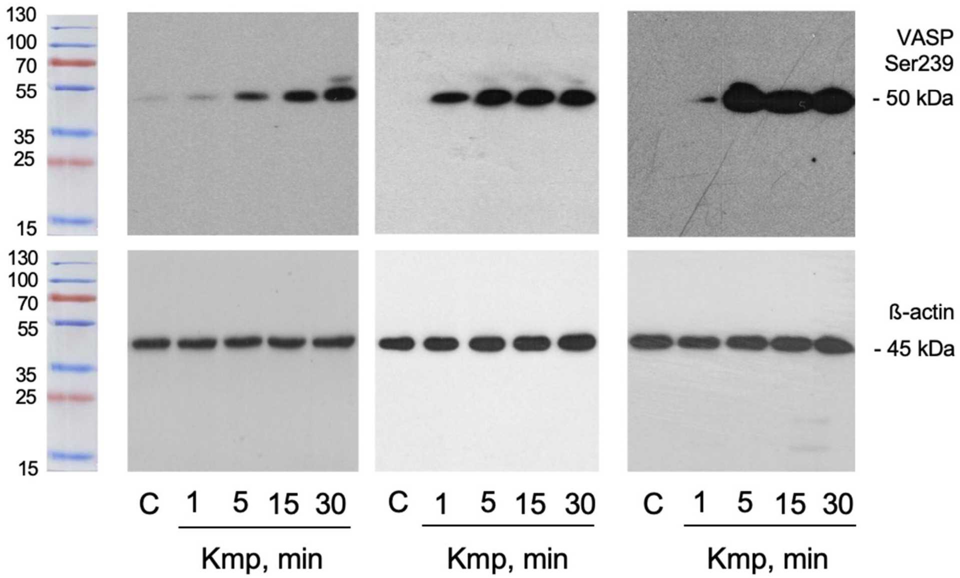

Figure S9
Full blots of figure 6f

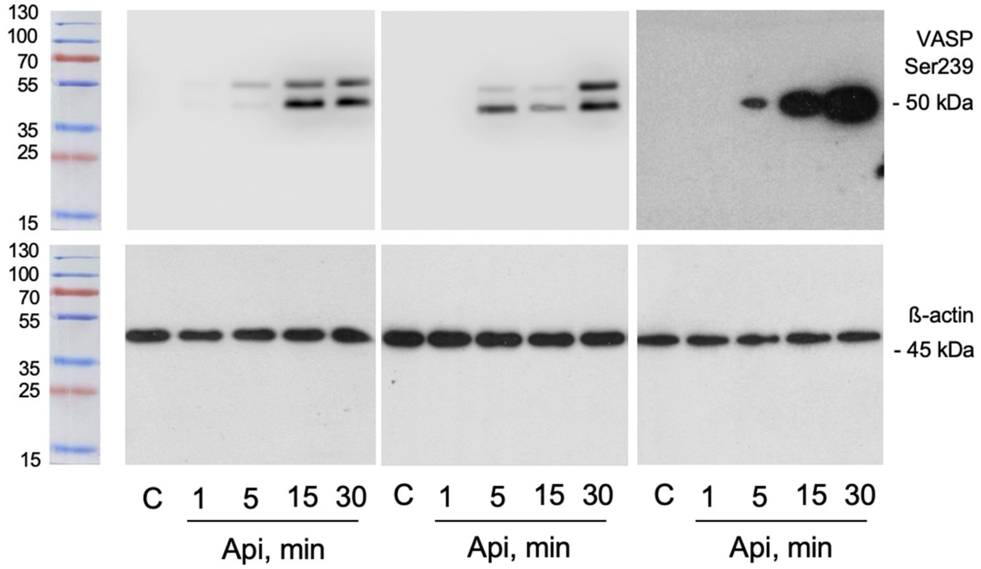

Figure S10
Full blots of figure 7a

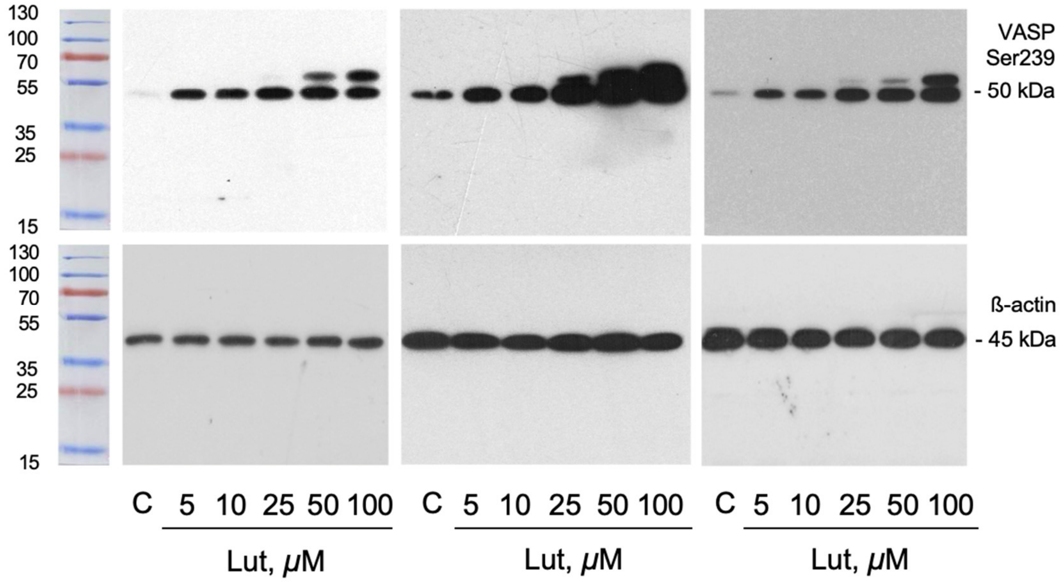

Figure S11
Full blots of figure 7b

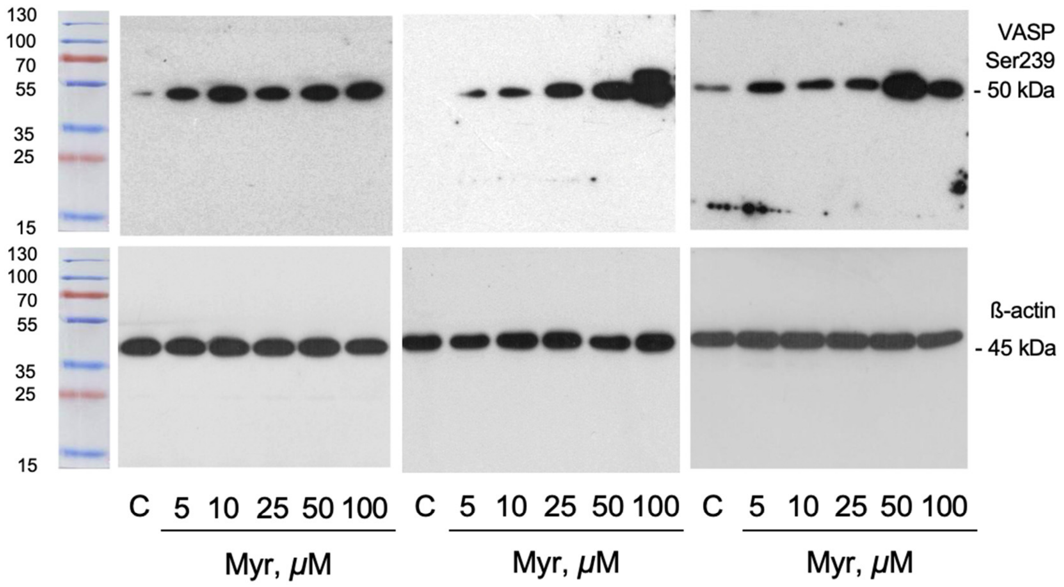

Figure S12
Full blots of figure 7c

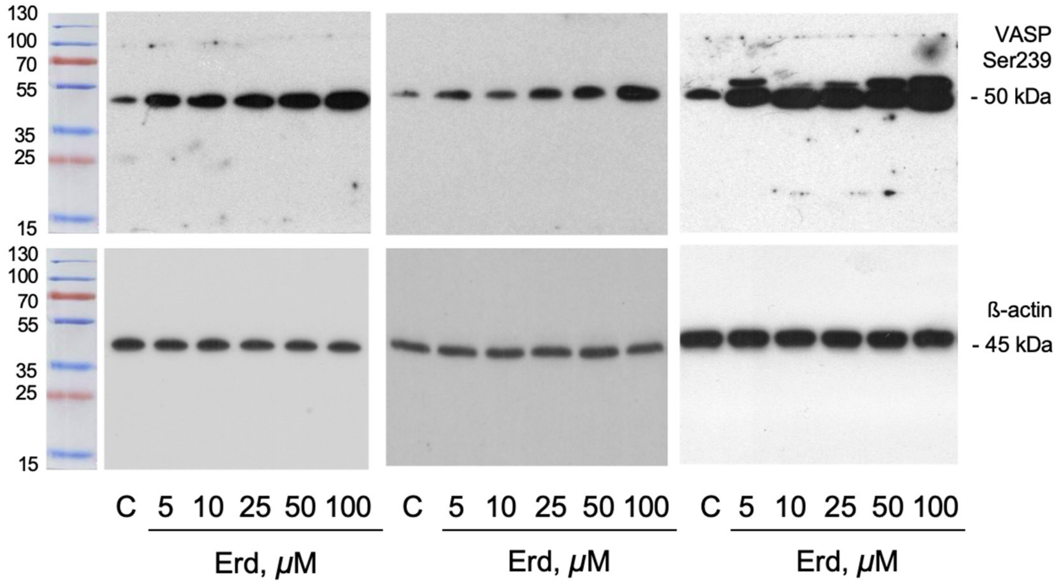

Figure S13
Full blots of figure 7d

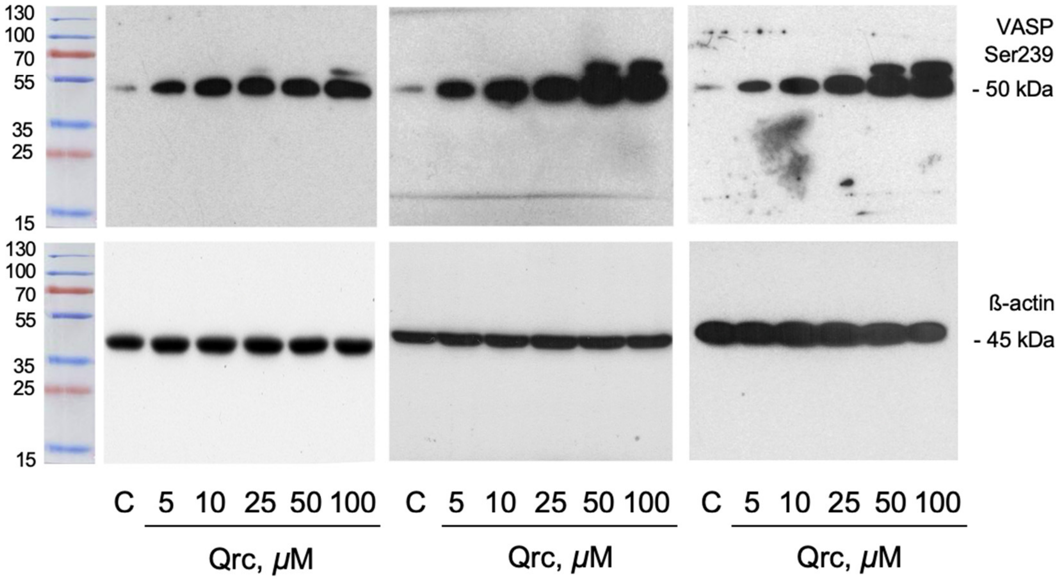

Figure S14
Full blots of figure 7e

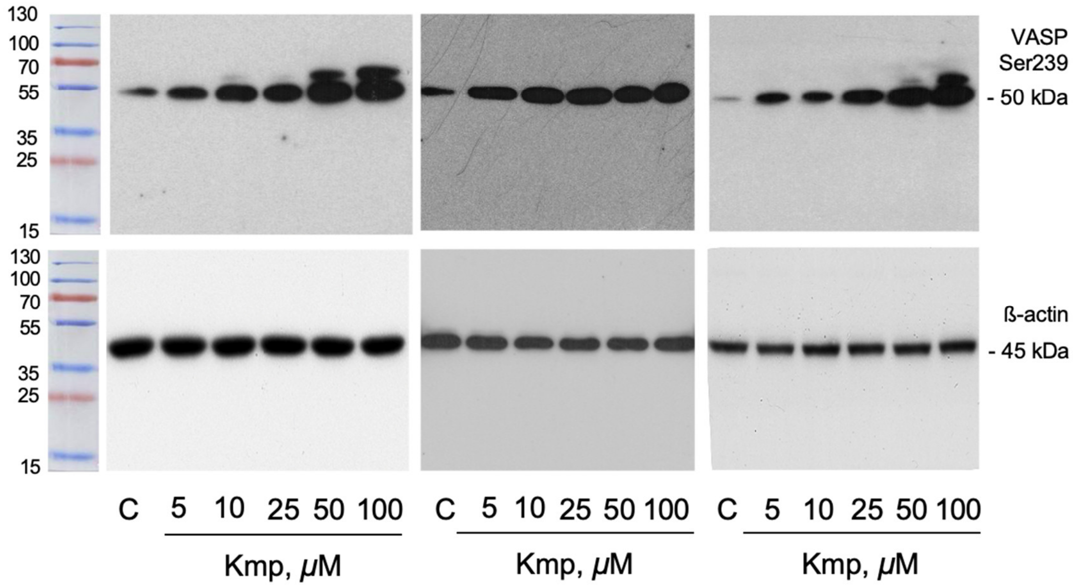

Figure S15
Full blots of figure 7f

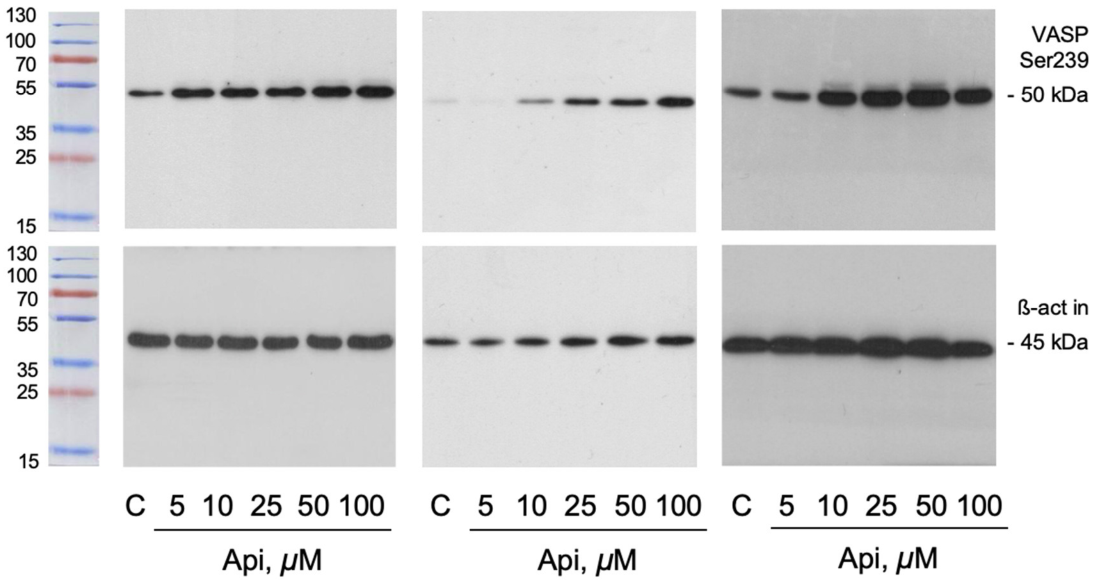

Figure S16: Full blots of figures 8 a-b

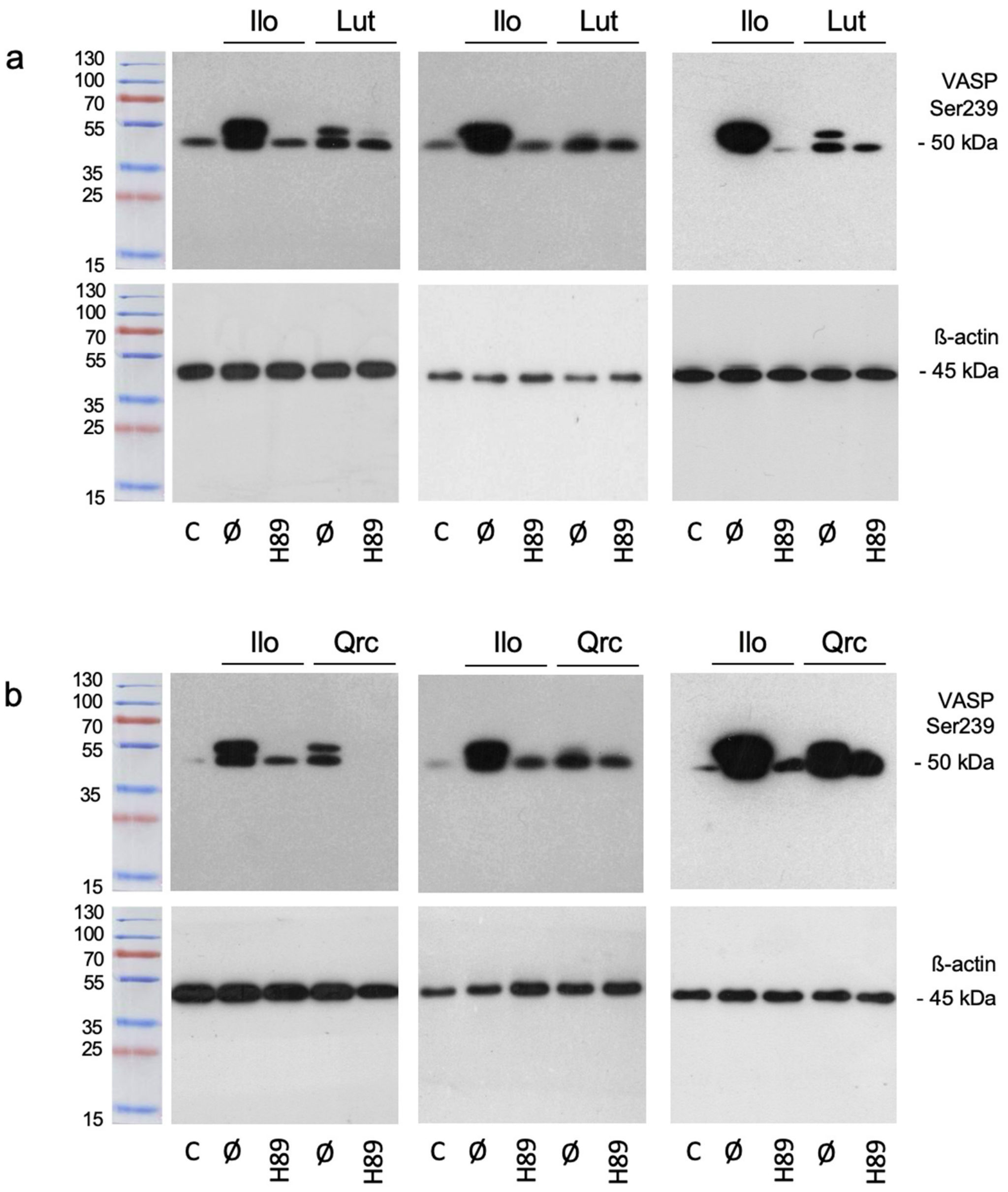

Figure S17
Full blots of figure 9a

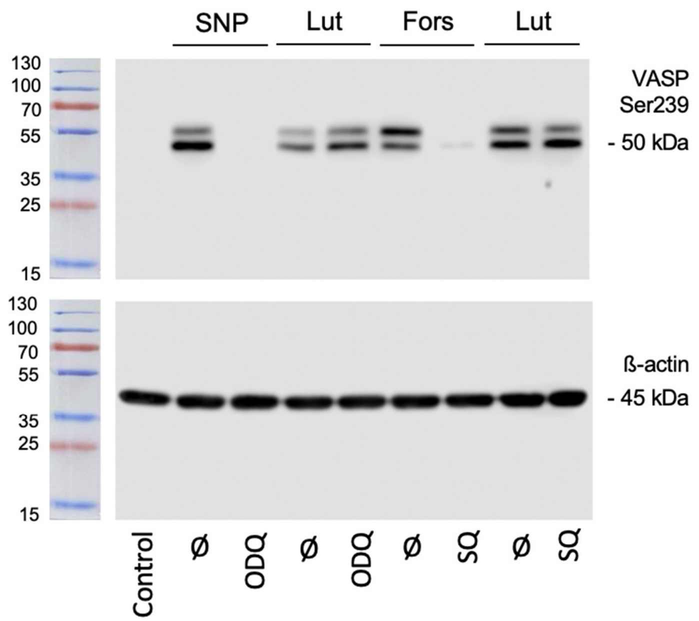

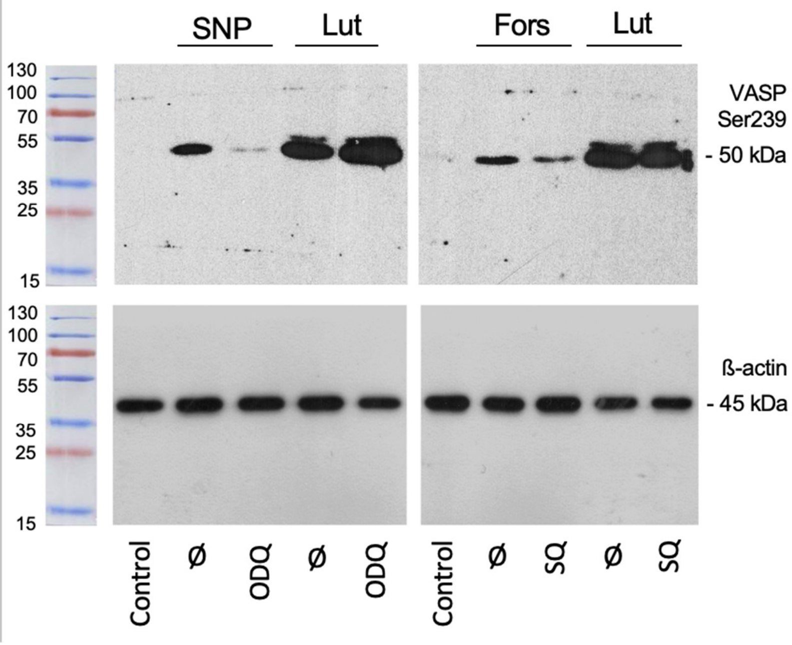

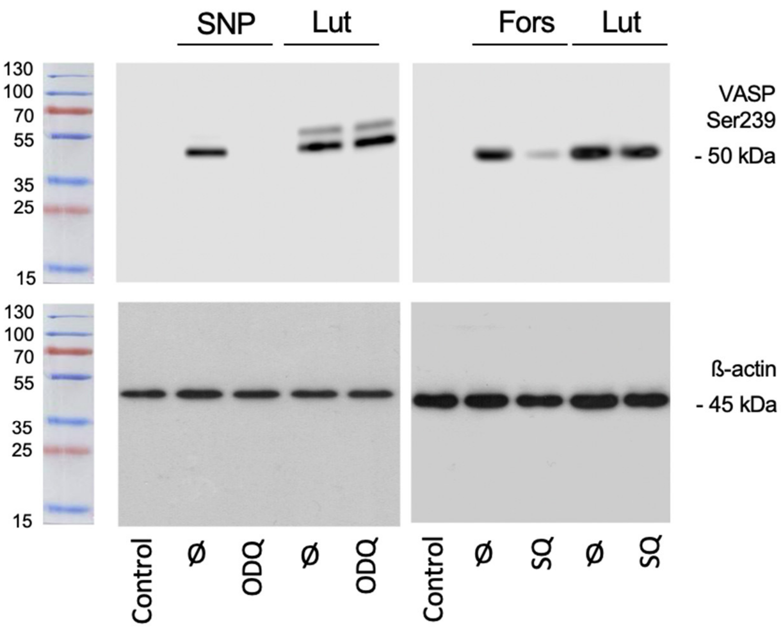

Figure S18
Full blots of figure 9b

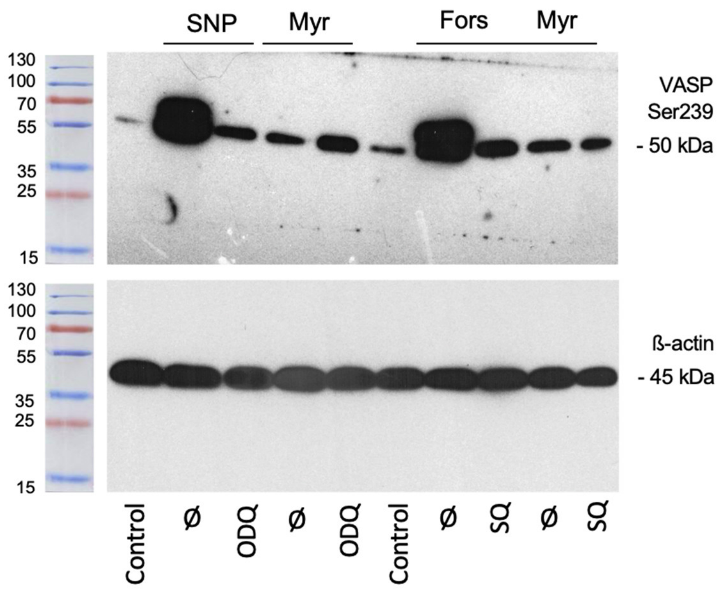

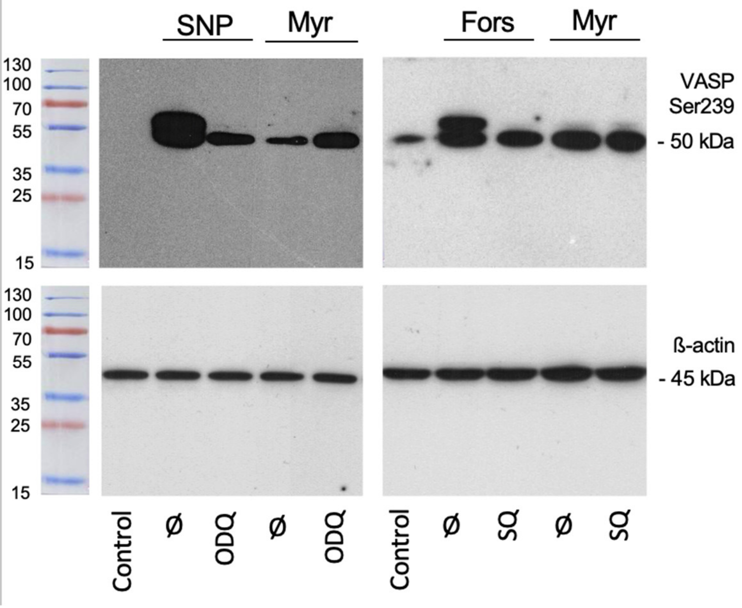

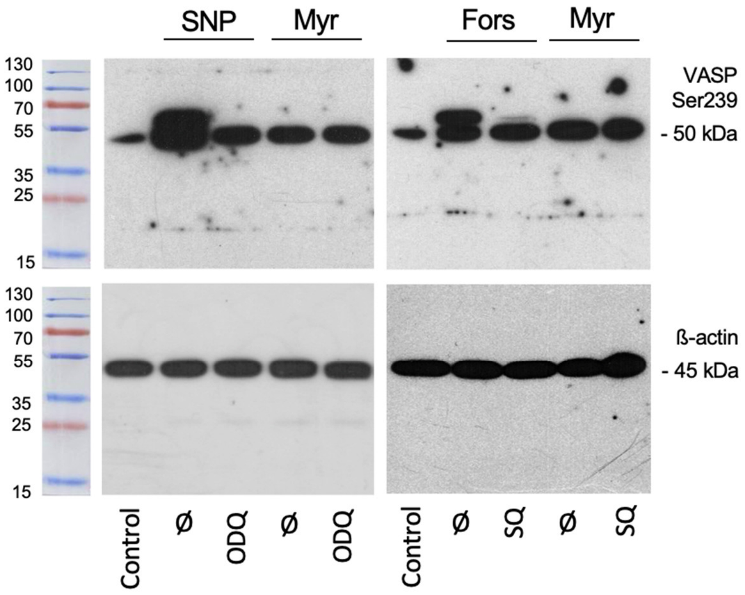

Figure S19
Full blots of figure 9c

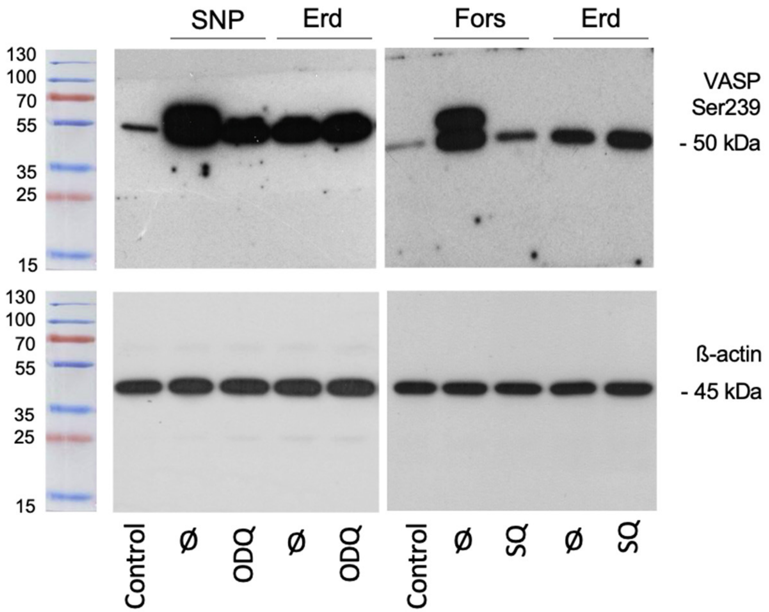

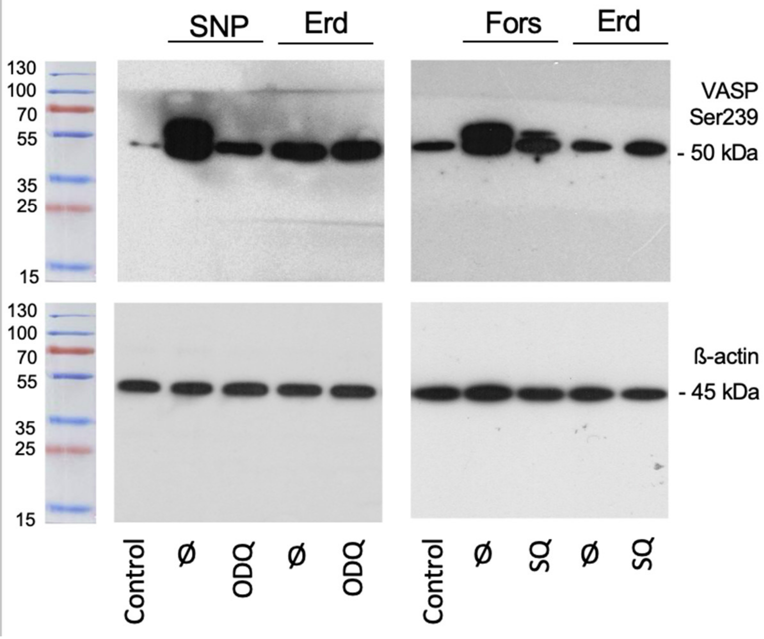

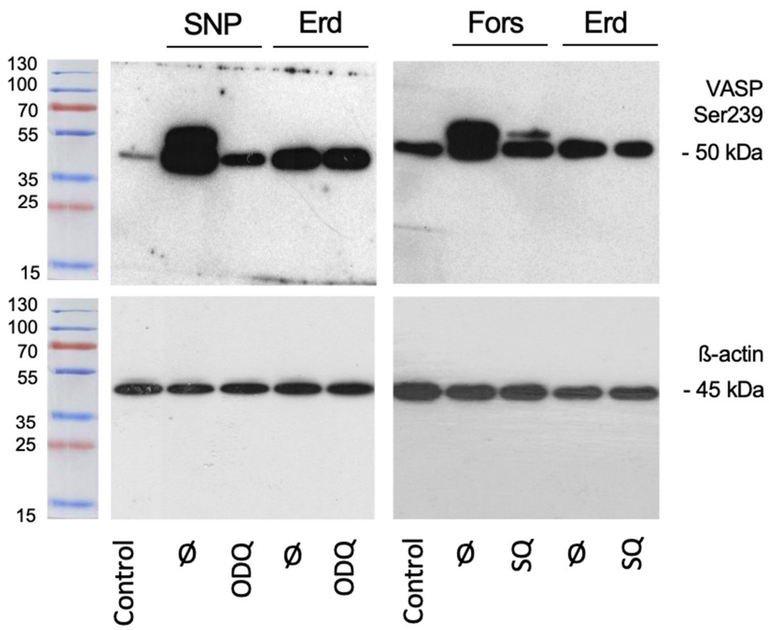

Figure S20
Full blots of figure 9d

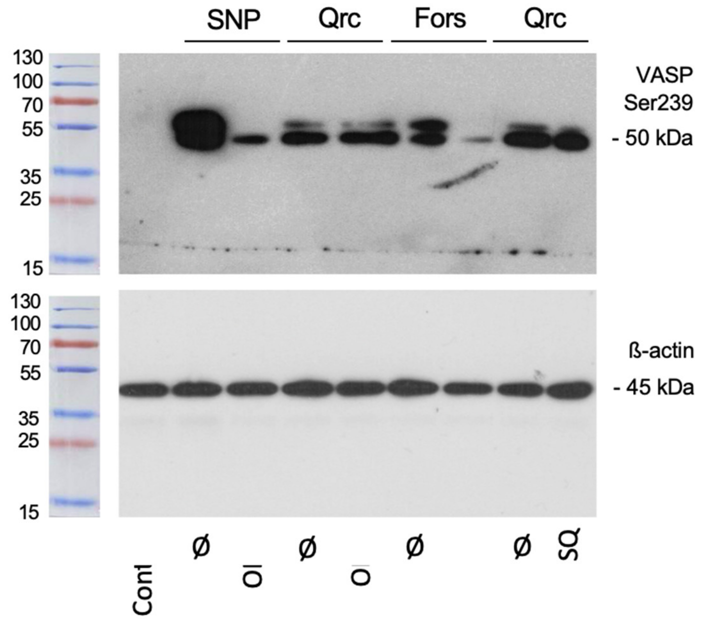

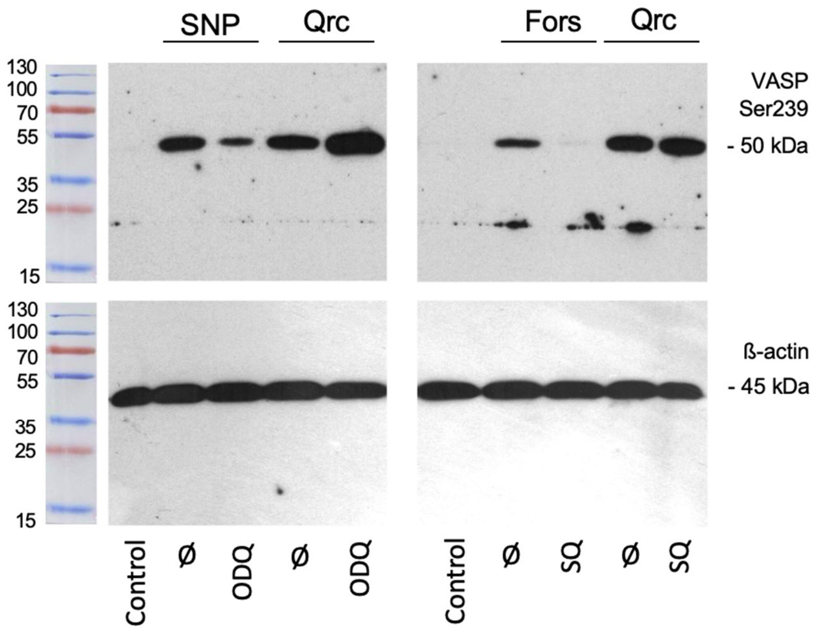

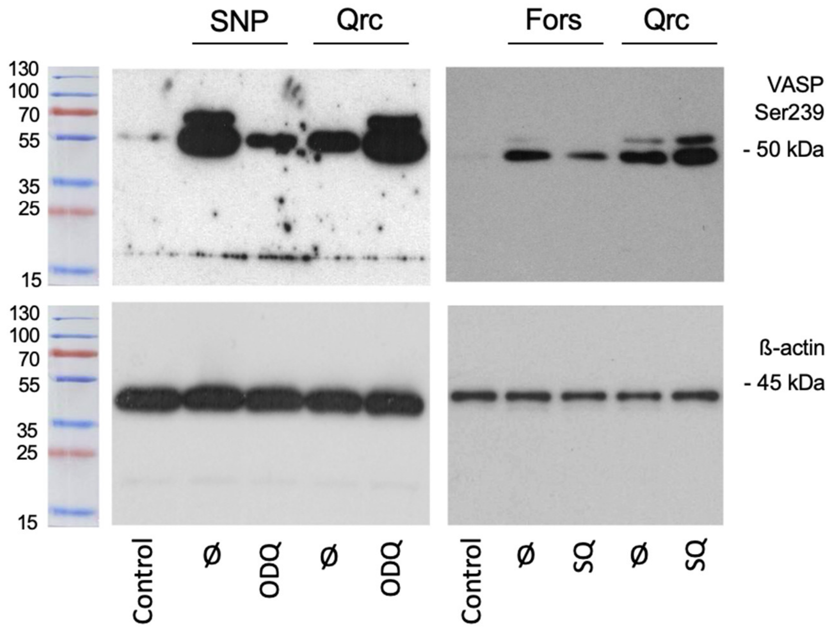

Figure S21
Full blots of figure 9e

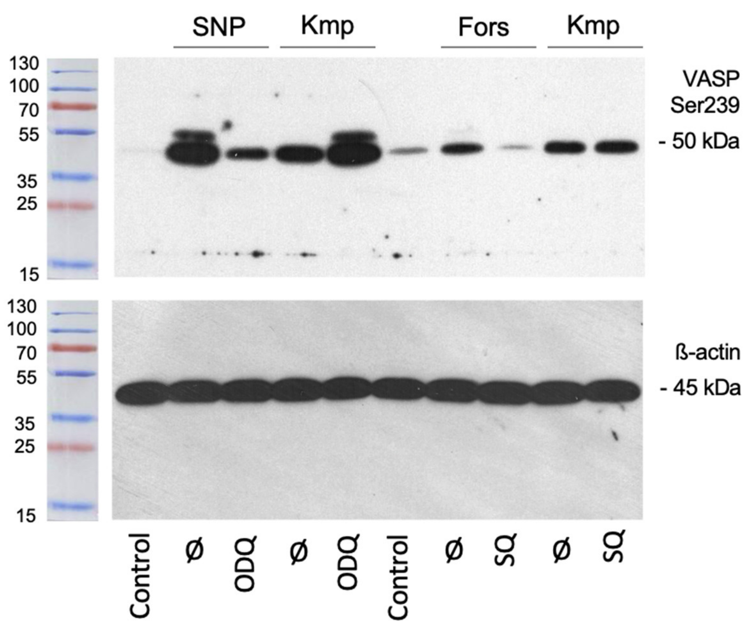

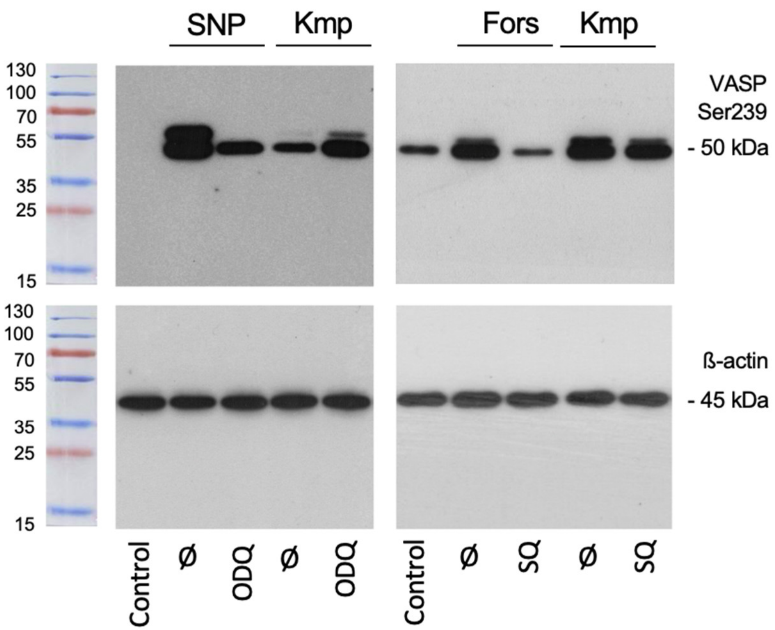

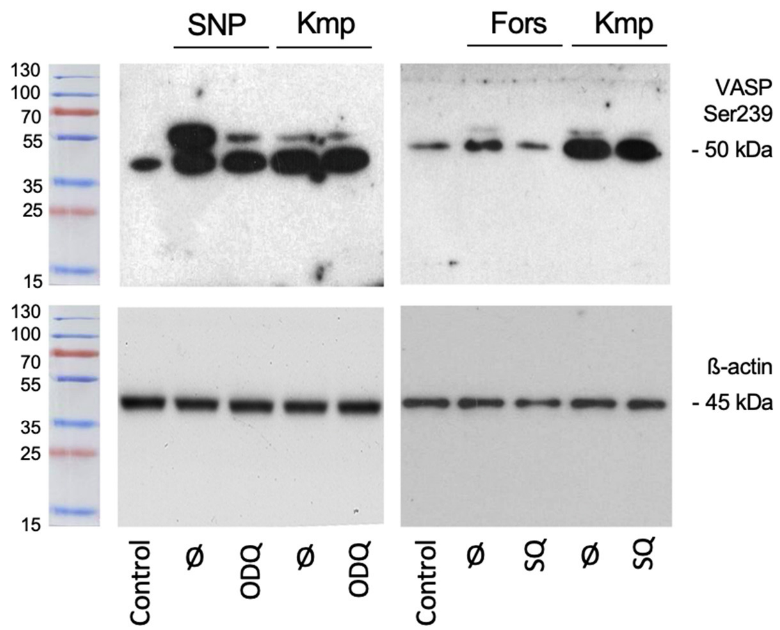

Figure S22
Full blots of figure 9f

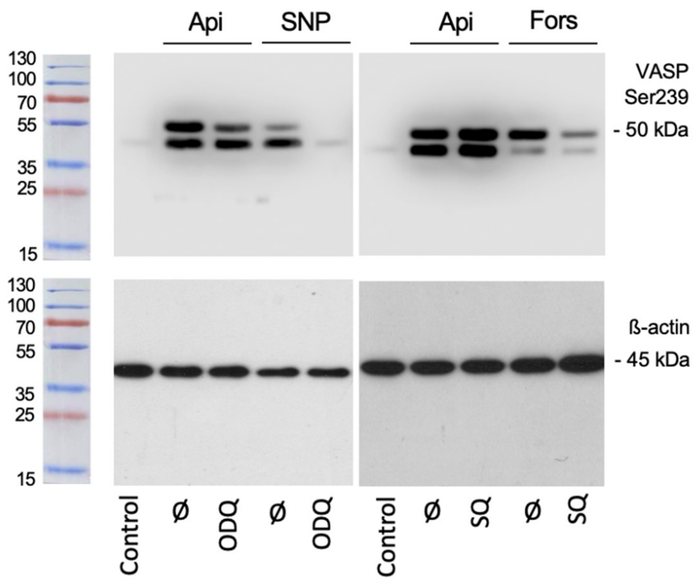

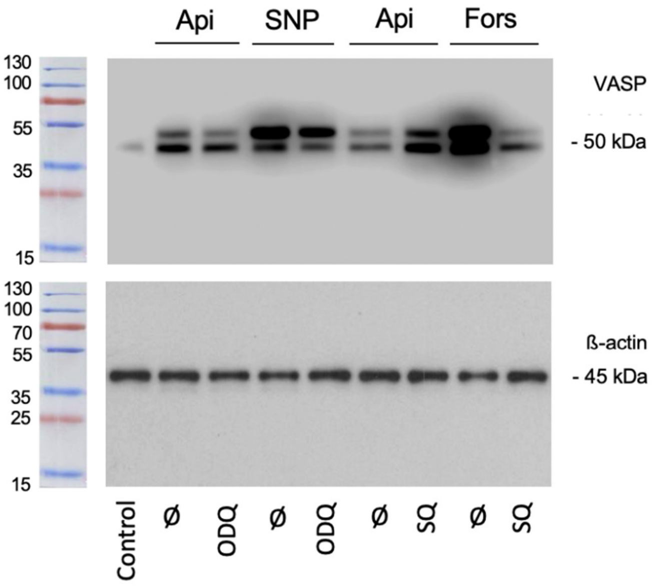

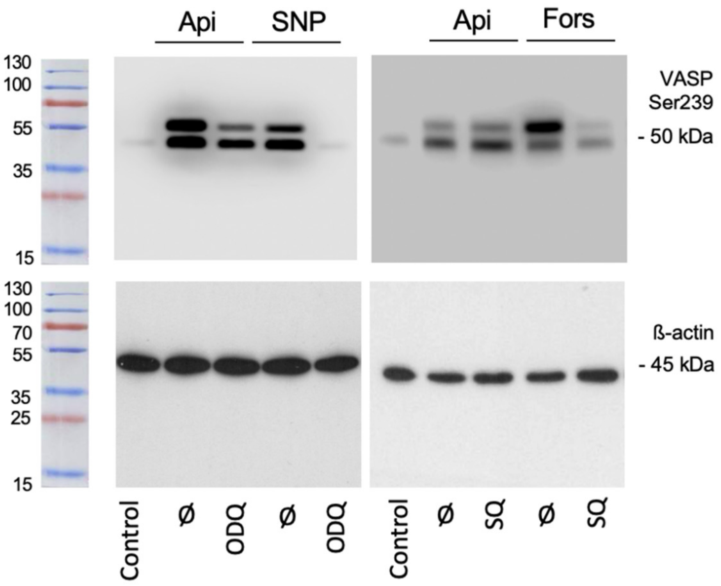

Figure S23
Full blots of figure 12

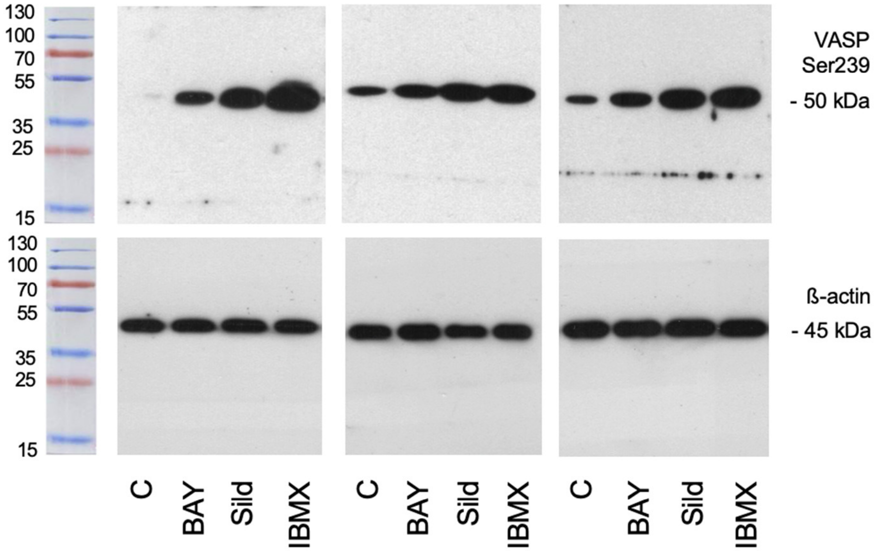

Supplement: Supplementary file 1 [file ijms-25-04864-s001.zip › ijms-2964889-supplementary.pdf]
